# Microbial community dynamics mediate rapid biomass loss and phosphorus recycling in pelagic *Sargassum*

**DOI:** 10.3389/fpls.2026.1918885

**Published:** 2026-10-05

**Authors:** Roberto Velázquez-Ochoa, Vivian Y. Li, Wiebke E. Krämer, Mónica Medina, Susana Enríquez

**Affiliations:** 1 Laboratory of Photobiology and Functional Allometry, Unidad Académica de Sistemas Arrecifales Puerto Morelos, Instituto de Ciencias del Mar y Limnología, Universidad Nacional Autónoma de México, Cancun, Mexico; 2 Department of Ecology and Evolutionary Biology, University of California, Los Angeles, Los Angeles, CA, United States

**Keywords:** algal decomposition, algal growth, algal-bacteria mutualisms, chemoheterotrophic bacteria, marine debris, microbial succession, nutrient cycling, Sargasssum blooms

## Abstract

*Sargassum* blooms in the tropical North Atlantic have surged to unprecedented levels, causing massive algal tides that severely impact coastal ecosystems. To improve the current understanding of *Sargassum* biomass dynamics and the contribution of this productive algal bloom to carbon and nutrient fluxes between the Atlantic and the Caribbean, we characterized the growth and decomposition rates of this pelagic alga, nutrient release rates during litter decay, and the bacterial microbiome at several stages of this decay. The analysis revealed no exceptional growth under increased nutrient availability but a rapid biomass mineralization with a litter half-life of 16 days, comparable to phytoplankton. This fast decomposition triggers swift nutrient release, notably phosphorus, which fuels coastal eutrophication and may help explain oxygen depletion and toxic gas emission during massive beaching events. A microbial succession involving chemoheterotrophic and nitrogen-fixing bacteria predicts functions involved in the biomineralization of nitrogen and phosphorus-containing compounds that may facilitate such efficient phosphorus mineralization, supporting similar patterns in marine debris to terrestrial plant litter, where nutrients drive stage-specific patterns in microbial community succession during litter decomposition. Our results underscore the pivotal role of microbial communities in enhancing algal resilience to nutrient deprivation—through the establishment of mutualistic associations with their host—and in mediating global carbon and nutrient fluxes, an aspect of particular relevance to the marine phosphorus cycle. Ultimately, this study emphasizes the severe ecological threat that massive *Sargassum* tides pose to marine health and public safety at the coast.

## 1 Introduction

A highly productive ecosystem has developed since 2011 in the North Equatorial Recirculation Region (NERR) (Winge, 1923), spanning approximately 5.5 million km² between South America and Africa (Wang et al., 2019), where it is seasonally aggregated by the intertropical convergence zone (ITCZ; Johns et al., 2020). Since 2011, it has continued to expand (Jouanno et al., 2023), reaching a historical record of 38 million tons in July 2025 (Optical Oceanography Lab, 2025; *Sargassum* outlook, 2025). While the origin and main drivers of this massive algal presence remain under discussion (Wang et al., 2019; Jouanno et al., 2023; Marsh et al., 2023; Jung et al., 2025), nutrient enrichment and global warming have emerged as two key contributors (Glibert et al., 2018; Velázquez-Ochoa et al., 2026). Nutrients are fundamental for triggering algal blooms (Glibert et al., 2018), and recent findings have documented a metabolic adaptation to heat in two genetic variants (Velázquez-Ochoa et al., 2026), which improves *Sargassum* photosynthetic performance and productivity at lower latitudes and in a warming ocean (Esper et al., 2024).

The average rates of photosynthesis (6–13 mg O_2_ g DW^-^¹ h^-^¹) and growth (0.04 - 0.06 d^-^¹) of pelagic *Sargassum* (Velázquez-Ochoa et al., 2026) fall within the mean range reported for macroalgal photosynthesis (~10 mg O_2_ g DW^-^¹ h^-^¹) (Enríquez et al., 1995) and growth (0.065-0.07 d^-^¹) rates (Nielsen et al., 1996) and below the rates of highly productive invasive species (≈0.077 d^-^¹) (Stiger-Pouvreau et al., 2023). The widely recognized diazotrophic activity for the microbiome of pelagic *Sargassum* (Parr, 1939; Venter et al., 2004; Rooker et al., 2006; Mendonça et al., 2007; Léger-Pigout et al., 2024; Cox et al., 2025) can also explain their resilience to starvation and why these floating populations can increase their growth and photosynthesis with experimental addition of P but not N (Lapointe, 1986). Large differences between genetic variants have been recently recognized for their physiology (Velázquez-Ochoa et al., 2026) and historically documented for *Sargassum* growth associated with their differential response to nutrients, temperature, and salinity tolerance (see the compiled literature in Velázquez-Ochoa et al., 2026). High growth rates relative to the estimated average have only been reported for one genetic variant, *S. fluitans* var. *fluitans* (former *S. fluitans* III), under nutrient-enriched conditions (Hanisak and Samuel, 1987; Leemans et al., 2025). Unfortunately, the important experimental shortcomings identified in the literature growth data (cf., Velázquez-Ochoa et al., 2026) call into question the published values, uncovering an important gap of knowledge in the biology of pelagic *Sargassum*.

The aim of this study is to shed light on this question by characterizing two traits intimately involved in algal biomass dynamics, *Sargassum* growth and decomposition rates. Since substantial nutrient levels are required to trigger highly productive algal blooms (Glibert et al., 2018; Lapointe, 1995) and large amounts of nutrients are released when *Sargassum* reaches the beach and accumulates in large quantities along the shore (Velázquez-Ochoa and Enríquez, 2023), the analysis also investigates the bacterial community responsible for biomass mineralization and nutrient release under experimental conditions. This information will help improve the current understanding of the contribution of this productive algal bloom to carbon and nutrient fluxes in the Atlantic and the impact of *Sargassum* tides on primary production, water quality, and the trophic condition of coastal ecosystems (Schultz and Urban, 2008; Dugan et al., 2011; Rodil et al., 2019). The analysis estimated a detritus half-life of 16 days, supporting fast biomass and nutrient cycling and uncovering an extraordinary *Sargassum* trait for biomass decomposition (Enríquez et al., 1993). This trait can contribute to explaining oxygen depletion and toxic gas emissions during massive algal tides (Rodríguez-Muñoz et al., 2021), highlighting the threat of eutrophication that they represent to coastal ecosystems. The study also provides insight into the role that mutualistic associations between algae and their microbiome may play in improving algal performance and resilience to starvation.

## 2 Methods

### 2.1 Sample collection

Fresh samples of pelagic *Sargassum* were collected in a Mexican Caribbean reef lagoon, Puerto Morelos (20° 52’ N, 86° 50’ W), before algal arrival on the beach. The annual average temperature of the lagoon water was previously documented to be at 28 °C, with an average annual maximum in August of 30 °C (Scheufen et al., 2017). The selected samples for experimental analysis were cleaned of epiphytes and epibionts and placed in 150L tanks with two underwater pumps that allowed maintenance of constant water circulation. The irradiance was shaded with neutral meshes and maintained at 80% of the surface irradiance (Es).

### 2.2 Growth experiments

Samples of *Sargassum fluitans* var. *fluitans* photoacclimated to the experimental conditions (single light treatment, two nutrient treatments, and three temperature treatments) were placed in beakers filled with 500 mL filtered seawater and bubbled with oxygen during the incubation, which were located in 150 L tanks with temperature control (**Supplementary Figure S1**; Scheufen et al., 2017). Oxygen bubbling allowed generating turbulence and breaking the boundary layer around algal tissues. Four beakers per tank and four/three tanks per treatment were used to perform three experiments. The first in August 2015, the second in August 2018, and the third in April-May 2020. In the first experiments, samples were maintained for 12 days under two contrasting nutrient conditions using: i) oceanic oligotrophic water from the Yucatan current (2.16 µM DIN and 0.21 µM DIP) (Sánchez-Quinto and Falcón, 2019); and ii) seawater collected from the reef lagoon with significantly higher nutrient concentrations (19.1 ± 4.14 µM DIN and 0.78 ± 0.1 µM DIP) (Velázquez-Ochoa and Enríquez, 2023). Therefore, 16 beakers (4 per tank) were analyzed per treatment with a total of 32 beakers. In the temperature experiment, samples were maintained in five replicates (beakers) per tank and three tanks per treatment under three temperature conditions (26 °C, 28 °C, and 30 °C). This resulted in 15 beakers per treatment and a total of 45 beakers analyzed (15 x 3). Changes in biomass gains and losses were measured after sample exposure for 32 days to these temperature treatments. The initial fresh weight of the samples of the first experiment was 0.44 g FW ± 0.07 (STD) per replicate with an average length of 5.19 ± 0.59 cm and approx. 15 ± 2 nodes per individual. A greater initial biomass of 4.8 g ± 0.7 g was used in the second experiment, while in the third, small samples of 0.55 g ± 0.06 (mean ± STD) and an average length of 8 cm ± 0.05 were again used. The small sample size and the selection of young segments were critical for employing homogenous samples in age and photoacclimatory condition (young thalli) and also for homogenizing the light environment within algal tissues during the incubation, minimizing thallus self-shading (**Supplementary Figures S1**, **S2**). Hence, by changing the sample size, we tested the potential interference of this variability on *Sargassum* growth estimations.

Thallus elongation rates, in mm per day, and node production, in number of nodes per day, allowed estimation of absolute growth after marking the initial samples and measuring the length, number of new nodes, and weight of the new growth at the end of the experiment. Relative growth rates (RGR, mg g^-^¹ d^-^¹) were estimated as


$$\left(LN\;\left(W_{f}\right)\;–\;LN\;\left(W_{i}\right)\right)/time\;\left(days\right),$$

where W_f_ refers to the final weight at day 12 or 32, and W_i_ to the initial weight at t=0. To quantify biomass loss during the experiment (W_BL_), we estimate first the amount of biomass that had to be present (W_est_) in the absence of biomass loss, as


$$W_{est}=\;W_{i}+\;W_{new}$$

and biomass loss (W_BL_) was quantified as the difference between this theoretical value and the final value observed (W_f_) at the end of the experiment, as follows:


$$W_{BL}=\;\left(W_{i}+\;W_{new}\right)\;-\;W_{f}$$

From these absolute values of biomass, we calculated the gross relative growth rates (gRGR) as a theoretical relative growth rate in the absence of biomass loss, as follows, where Δt refers to the number of days between the initial (t_i_) and final (t_f_) time:


$$gRGR\;=\;\left(LN\;\left(W_{i}+\;W_{new}\right)\;–\;LN\;\left(W_{i}\right)\right)/\Delta t$$

Finally, we also estimate the relative biomass loss rates (RBLR) assuming that biomass loss only affected the initial sample (no biomass loss in the new growth was detected during the incubation) as follows:


$$RBLR\;=\;\left(LN\;\left(Wi\;-\;W_{BL}\right)\;–\;LN\;\left(W_{i}\right)\right)/\Delta t$$

### 2.3 Decomposition experiment

Freshly collected samples of pelagic *Sargassum* from the ocean were placed in a 0.84 m³ tank (2 × 0.7 × 0.6 m). Pre-weighted samples were introduced in 10 homemade cylinders of flexible and dense plastic, which were open on both sides to facilitate monitoring fresh weight changes (i.e., biomass loss) at different time points along the experiment (**Supplementary Figure S3**). A “seed community” of microbial decomposers collected from the beach of the reef lagoon of Puerto Morelos was placed at the head of the tank, next to the freshly collected samples, but not in direct contact with them. This “seed algal detritus” was recently beached/dead material that had already initiated the decomposition process (**Supplementary Figure S3**). We only collected detritus with no signs of ongoing anaerobic processes. The biomass located in each of the 10 cylinders was weighed at specific times during the experiment. The cylinders were refilled with new material, previously weighted, from the detritus located in the tank, which was undergoing the same degradation process as the samples located in the cylinders. Removed samples were dried and preserved for further analyses, and a subsample was frozen at -80 °C for the microbiome analyses (see below). Samples in the tank were exposed to full solar irradiance, which allowed water evaporation and prevented the accumulation of water volume exudates within the tank. However, the detritus in the tank remained moist throughout the 20 days of the experiment.

### 2.4 Nutrient analysis

Detritus samples were dried at 60 °C for 48 h, then ground and homogenized in a mortar using a manual pestle. Powder samples were treated with 10% HCl to remove CaCO_3_ until the bubbles ceased and dried overnight at 60 °C (Kennedy et al., 2005). They were later rinsed with Milli-Q water to remove HCl residues and dried again overnight at 60 °C. Five milligrams of each sample were encapsulated in 5 mg tin capsules (5 × 9 mm) and sent for analysis at the University of California’s Stable Isotope Facility (SIF) for the determination of nitrogen (%N) content with an elemental analyzer (PDZ Europa ANCA-GSL). For technical details and the material references used, see https://stableisotopefacility.ucdavis.edu/. Phosphorus content (%P) of the samples was determined using high-resolution inductively coupled plasma mass spectrometry (HR-ICP-MS) (Thermo Scientific Element 2XR) at the Stable Isotope Laboratory of the ICML-UNAM in Mazatlán, Mexico. To facilitate the conversion of fresh weight to dry weight values, we compared direct measurements of both values for 32 samples. The average water content of pelagic *Sargassum* was ≈80% of its fresh weight, indicating that the dry weight represents approximately 20% of the fresh weight (FW = 0.2 ± 0.0023 * DW; R² = 0.93; p < 0.001; **Supplementary Figure S4**). Samples for N determinations were lost beyond day 15 of the decomposition experiment. Three different models then were used to estimate the %N remaining at the end of the decomposition experiment from the preceding %N values: a) assuming a linear change for the last 5 points (%N remaining = a + b*Day; n = 5); b) assuming an exponential decline for the 6 points directly estimated (%N remaining = a * e^b*Day^; n = 6); and c) using a power function for the last 3 points (%N remaining = a*Day^-b^; n = 3).

### 2.5 Microbiome characterization

Samples of *Sargassum* detritus for microbiome characterization were preserved in 95% ethanol and stored at -80 °C until processing. Microbiome DNA extraction was performed using the DNeasy Blood and Tissue Kit (Qiagen, Hilden, Germany; with RRID: SCR_008639) according to the manufacturer’s protocol. This enzymatic lysis may have failed to capture the full diversity of bacterial taxa present in the community, as no bead beating was performed in the DNA extraction, typically required to disrupt the peptidoglycan cell wall of gram-positive bacteria. gDNA concentration was quantified at 0.5-1.5 µg (Qubit^®^ dDNA BR Assay Kit on a Qubit^®^ 2.0 Fluorometer). DNA samples were transported to Dr. Medina’s laboratory, where the V4 region of the bacterial 16S rRNA gene was amplified in 20 µl PCR reactions following the Global Coral Microbiome Project (GCMP) amplicon sequencing protocol using the 515F/806R primer pair (Epstein et al., 2025) with Illumina IDT linkers attached to the 5’ end. PCR products were submitted to the Rush University Medical Center Genomics and Microbiome Core Facility for library preparation and paired-end sequencing on the Illumina MiniSeq platform. Data preprocessing, including primer and adaptor removal, trimming and filtering by sequence length, and filtering by sequence quality, was performed by the facility. Sequences received from the sequencing facility were processed and analyzed in R (version 4.4.1). All figures were created using ggplot2 (version 3.5.2.). QIIME 2 (Bolyen et al., 2019) was used for primer and adaptor removal. DADA2 v1.35.0 (Callahan et al., 2016) was used to trim and filter sequences by length, correct sequence errors, dereplicate sequences, merge forward and reverse overlapping reads, remove chimeras, infer amplicon sequence variants (ASVs), and assign taxonomy using the RDP Naive Bayesian Classifier algorithm (k-mer size 8, 100 bootstrap replicates) (Wang et al., 2007) and SILVA SSU database version 138. Assignments with bootstrap confidence values <80% were classified as “NA”. ASV tables were imported into R for downstream analyses using phyloseq v1.48.0 (McMurdie and Holmes, 2013). Sequences that mapped to mitochondrial or chloroplast 16S were removed. Sequencing of the V4 hypervariable region of the bacterial 16S rRNA gene is known to be limited in discriminatory power at lower taxonomic levels such as genus and species (https://pmc.ncbi.nlm.nih.gov/articles/PMC12367389/). Therefore, bacterial taxonomic assignment was performed only down to the Family level. The computational FAPROTAX tool (Functional Annotation of Prokaryotic Taxa; version 1.2.12) (Louca et al., 2016) was used to infer functional predictions for the microbiome community from 16S rRNA data. The PICRUSt2 (Phylogenetic Investigation of Communities by Reconstruction of Unobserved States; version 2.6.3; Douglas et al., 2020) was also used to infer KEGG ortholog (KO) functional profiles for the microbiome community from 16S rRNA data.

### 2.6 Data analysis

Values of the experimental results are expressed as the mean ± standard error (SE) of the data measured. A one-way ANOVA test was used to compare values among experimental treatments. Tukey *post hoc* tests enabled the identification of significant differences among species and/or treatments. Kruskal-Wallis tests were used to evaluate significant differences between phases in the bacterial community. The data for the growth and decomposition experiments were analyzed using R-software (R Core Team, 2020) and Kaleidagraph software.

Relative abundances of bacterial groups were log-ratio transformed using the R package compositions v2.0-9 (van den Boogaart et al., 2025). A pseudo-count of 1 was added to all OTU counts to enable log-ratio transformation of zero values. Principle coordinate analysis (PCoA) and non-metric dimensional scaling (NMDS) plots were generated using ggplot2 (Wickham, 2016) and used to visualize community dissimilarities with Aitchison distances using phyloseq v1.48.0 (McMurdie and Holmes, 2013). Analysis of similarities (ANOSIM) and Permutational Multivariate Analysis of Variance (PERMANOVA) were performed to test for significant differences in microbiome composition between sequencing runs using the R packages vegan v2.7-1 (Oksanen et al., 2025), veganEx v0.1.0 (GitHub, 2021) and RVAideMemoire v0.9-83-7 (Hervé, 2025), with 999 permutations per test.

Alpha-diversity matrices were calculated using vegan v2.7-1 (Oksanen et al., 2025). Kruskal-Wallis tests were run using the stats package version 4.5.1 to recognize significant differences in alpha diversity between the three microbial communities identified and those that defined three phases in the decomposition process, while *post-hoc* pairwise Wilcoxon tests were performed using the same package to identify significant pairwise differences between these three decomposition phases. Functional predictions of microbial communities were inferred from 16S rRNA data with FAPROTAX version 1.2.12 (Louca et al., 2016). Kruskal-Wallis significance tests were run using the stats package version 4.5.1 to identify functions and taxa that were significantly different between decomposition phases. Differential abundance analysis for PICRUSt2 predictions was performed using the R package ggpicrust2 (version 2.5.2) (Yang and Zhang, 2023) with the LinDA method (Zhou et al., 2022) and adjusted for false discovery rate using the Benjamini-Hochberg (BH) procedure.

## 3 Results

### 3.1 Growth rates

Three experiments were conducted to estimate the growth rates of *Sargassum fluitans* var. *fluitans*: two at contrasting nutrient availabilities and its optimal temperature (30 °C) (Velázquez-Ochoa et al., 2026) and a third to evaluate its response to temperature (**Figure 1**). Samples in the first experiment, August 2015, were maintained for 12 days under two nutrient conditions using oligotrophic oceanic water from the Yucatan current (2.16 µM DIN; 0.21 µM DIP) (Sánchez-Quinto and Falcón, 2019) and seawater from the reef lagoon with higher nutrient concentration (19.1 µM DIN; 0.78 µM DIP) (Velázquez-Ochoa and Enríquez, 2023). Young thalli with a fresh weight of 0.44 g ± 0.07 (STD), an average length of 5.19 ± 0.59 cm, and 15 ± 2 nodes per individual were selected to ensure homogeneity in age and the photoacclimatory condition between samples. This approach also helped to homogenize the light environment of algal tissues during the incubation, minimizing self-shading (**Figure 1A**; **Supplementary Figure S1**). Thallus elongation rates (mm d^-^¹) and relative growth rates (RGR, mg g DW d^-^¹) did not differ between treatments (t-test, p>0.05), but node production presented significant differences (**Figure 1B**). Testing for significant differences from 0 in the RGR estimations, we found positive growth for the treatment with higher nutrient supply (t-test, p<0.05) but no differences for the treatment with oligotrophic oceanic water (t-test, p>0.05), uncovering substantial biomass losses during the incubation in five beakers of the treatment with oligotrophic oceanic water for thalli that were elongating at almost similar rates in both treatments, although with lower node production under lower nutrient supply (**Figures 1C, D**).

**Figure 1:**
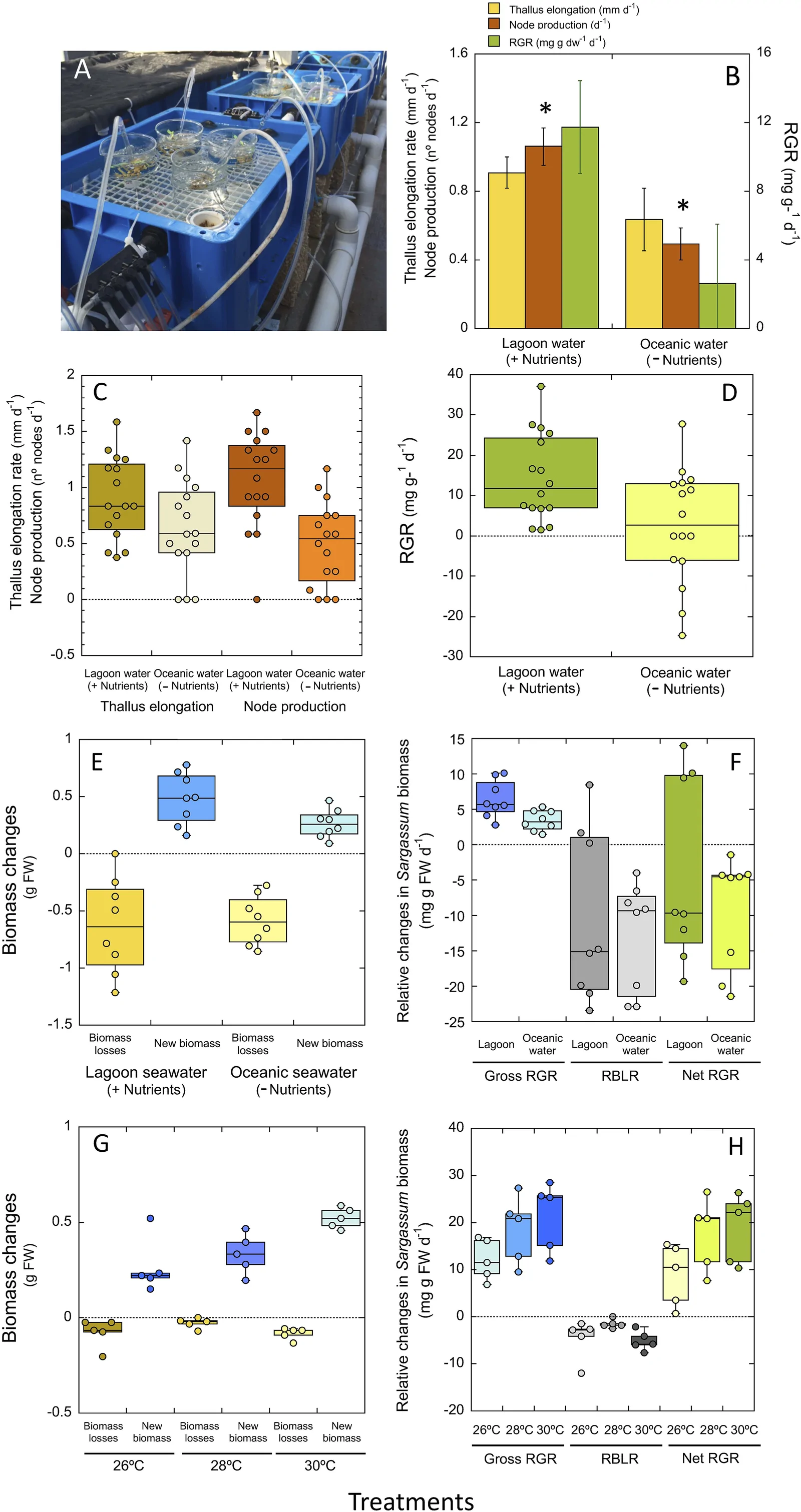
Experimental estimates of *Sargassum* growth rates. **(A)** Experimental setup: In the first and second experiments, four tanks per treatment and four replicates per tank (n=16 per treatment) were used to determine the growth rates of *Sargassum fluitans* var. *fluitans* under two nutrient treatments. In the third experiment, three tanks and five replicates per tank were used to investigate the differential response to temperature (n=15 per treatment). Samples were bubbled with oxygen during the incubation, and neutral meshes allowed 20% reductions in solar natural illumination. The image corresponds to the first experiment, and Plots B, C and D illustrate the results of this experiment: **(B)** Average ± SE values at day 12 for absolute growth (elongation rates, mm d^-^¹), node production (# nodes produced per day), and relative growth rates (RGR, mg gDW^^-^¹^ d^^-^¹)^ (n=16 per treatment and an initial sample size of 0.44 g FW ± 0.07); and **(C)** and **(D)** Box plots illustrating the variability observed among replicates for each nutrient treatment. Significant differences between the two nutrient treatments are indicated in plot B with an asterisk (elongation rates = 0.91 ± 0.09 vs. 0.64 ± 0.11 mm d^-1^; t-student, p>0.05, df=31). Plots **(E)** and **(F)** illustrate the results of the second experiment at day 12 (n=16 per treatment and an initial sample size of 4.8 g FW± 0.7 g) for the variation of biomass losses and new growth (g FW) and their relative changes: relative growth rates assuming no biomass losses (Gross RGR or gRGR, mg g DW^-^¹ d^-^¹); relative biomass loss rates (RBLR, mg g DW^-^¹ d^-^¹); and net relative growth rates (Net RGR, mg g DW^-^¹ d^-^¹). Plots **(G, H)** illustrate these results for the third experiment at day 32 (n=15 per treatment and an initial sample size of 0.55 g FW ± 0.06). Box plots encompass the 25 and 75% quartiles, the central solid line corresponds to the median, and open circles indicate values beyond the quartiles.

Two additional experiments were conducted to further quantify this biomass loss, one in August 2018 using similar treatments but larger samples (4.8 g FW ± 0.7; **Supplementary Figure S2**) and a third experiment in April-May 2020, where again only small, young samples (0.55 g FW ± 0.06, mean ± STD) were used and exposed to three temperature treatments (**Figures 1E–G**). Significant variability in new growth and biomass loss was observed between treatments, highlighting the interference of *Sargassum* biomass loss on the growth rate determinations. When algal growth was corrected for biomass losses (gross RGR or gRGR), the differences between treatments were easier to discern (**Figures 1F, G**). The fastest growth and largest biomass loss were estimated for samples exposed to the highest temperature and nutrient availability (**Figures 1E, F**). However, the suboptimal variability among replicates (STD = 18.4 mg g^-^¹ d^-^¹) observed in the second experiment strongly affected the reliability of the estimated values, questioning the conclusion, inconsistent with the first experiment, that larger biomass loss occurs at higher nutrient availability. When homogeneous young samples were selected (first and third experiments), this variability significantly declined (**Figures 1G, H**).

### 3.2 *Sargassum* decomposition rates and detritus nutrient release

A new experiment was conducted to investigate this fast biomass loss during growth. Freshly collected samples in the ocean were placed in a 0.84 m³ tank (2×0.7×0.6 m), where recently beached algal debris was also separately positioned at the head of the tank to help the new algal biomass to be colonized by microbial decomposers. ‘Cleaner’ experiments were previously performed without this algal debris (see **Supplementary Figures S3A, B**), but their lack of success evidenced the fundamental contribution of this ‘seed microbial community’ to facilitate the decomposition process in the tank. *Sargassum* was maintained for 20 days under these experimental conditions, exposed to full solar radiation, and the remaining algal debris was weighed at 14 time points. Litter biomass decay allowed the estimation of an exponential decomposition rate of -0.128 ± 0.013 d^-^¹ with a detritus half-life of 16 days (**Figure 2A, B**).

**Figure 2:**
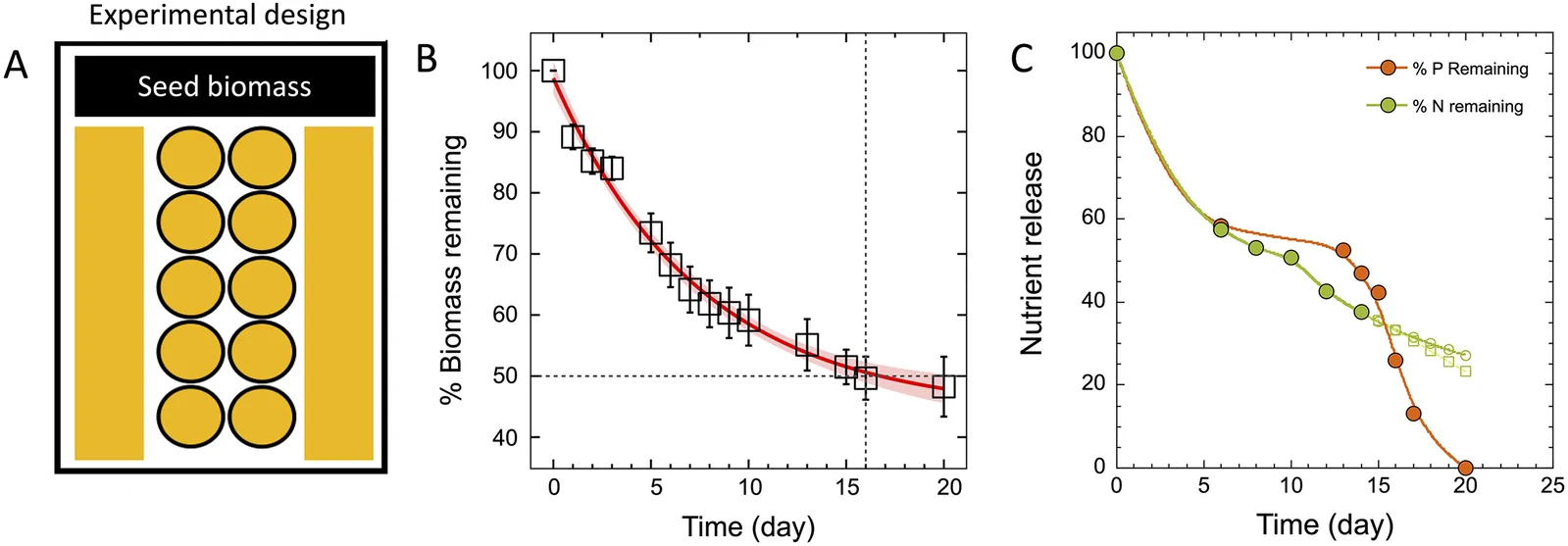
Experimental determination of the decomposition rate of pelagic *Sargassum*. **(A)** Scheme of the experimental design. **(B)** Mean ± SE (n=10) for the biomass decline of *Sargassum* detritus at specific time points of the experiment, quantified as percentage of fresh weight remaining. The red line describes the exponential function [y = a*exp(-b*x + c) + d] that fits the values, according to the equation: % Detritus remaining = 4 *exp (-0.128 * Time + 2.63) + 42.9 [R^2^ = 0.99; p<0.001]. The discontinued horizontal and vertical black fine lines indicate the number of days required for the disappearance of 50% of biomass detritus (half-life of *Sargassum* litter). **(C)** Percentage of nitrogen (%N) (in green) and phosphorus (%P) (in orange) remained in the *Sargassum* detritus along the decomposition process. A green discontinuous line and open green circles and squares illustrate the predicted % of nitrogen remaining in *Sargassum* detritus until day 20, assuming three models [linear (circles): %N remaining = -2.5 + 76.29 * Day; (R^2^ = 0.97; n = 5); exponential: %N remaining = 94.31 * e^-0.067*Day^ (R^2^ = 0.99; n= 6); and a power fit (squares): %N remaining = 396.15 * Day^-0.894^ (R^2^ = 0.999; n=3)]. Only the values for two models (linear and power function) are shown, as the values predicted by the exponential model were in between them. The linear model (circles) predicted 23% N remaining by day 20, the exponential model 24.8%, and 27% was predicted by the power function (squares).

Nutrient release during *Sargassum* decomposition was assessed by measuring the remaining percentages of nitrogen (N) and phosphorus (P) in the algal detritus. In the first 6 days, almost 40% of P was released, while 62.5% of N did in the first 14 days. Between days 6 and 13, P release was minimal (5%) and turned again to a rapid release by day 13, resulting in 87% loss by day 17 and 100% loss by day 20 (**Figure 2C**). Nitrogen mineralization, initially faster, showed a non-linear and, thus, slower decline pattern than phosphorus, consistent with the larger increases in dissolved inorganic P (DIP) reported during massive *Sargassum* tides relative to nitrogen (DIN) in both the water column and sediments (Velázquez-Ochoa and Enríquez, 2023). However, the samples after day 15 were, unfortunately, lost, and in order to compare the patterns of N and P decline, %N values were predicted using linear, exponential, and power functions from the preceding data. The values estimated for %N remaining in *Sargassum* litter by day 20 were similar for the linear and the exponential models (≈23-24.8%) and slightly higher for the power model (≈27%) (see **Figure 2C**). These values cannot be considered real estimates of the %N remaining in *Sargassum* detritus but indirect estimates assuming that no other processes may accelerate or slow down the pattern of decline predicted from the previous measurements.

### 3.3 Bacterial community of *Sargassum* detritus

To shed light on this rapid mineralization and nutrient release, the bacterial microbiome was characterized at eight different time points during the decomposition process. Three distinct clusters (**Figure 3A**; PERMANOVA and ANOSIM tests; p <0.05; see **Table 1**) highlighted three possible phases in the decomposition process: early, middle, and late (**Figure 3B**). Contrary to previous findings (Mendonça et al., 2024), no dysbiosis was detected. Species richness increased significantly from the early to the middle phase (**Figure 2C**; p = 0.0049, pairwise Wilcoxon) and remained constant through the late stage. Shannon’s and Simpson’s diversity indices continued increasing between middle and late phases (**Figures 3D, E**), although differences were not significant (p > 0.05, pairwise Wilcoxon).

**Figure 3:**
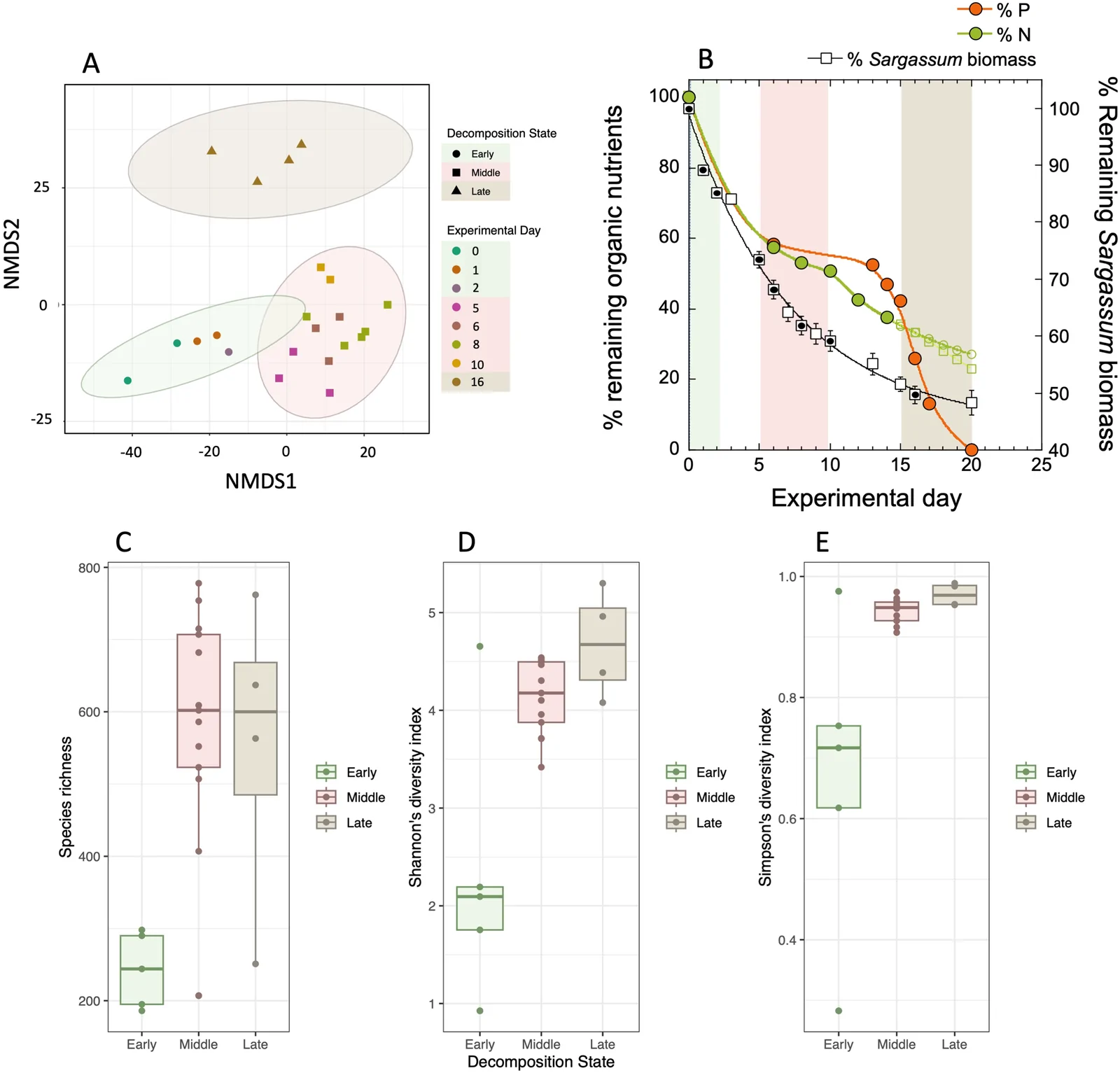
Variation of the bacterial communities during the decomposition process. **(A)** Describes the three clusters found using a non-metric dimensional scaling test (NMDS, stress = 0. 08060933) based on Aitchison distances between experimental days for samples characterized by days 0, 1, 2, 5, 6, 8, 10, and 16 during the decomposition process. Pairwise PERMANOVA and ANOSIM tests support significant pairwise differences between clusters. Ellipses represent 95% confidence intervals for each cluster: green for the early phase, pink for the middle phase, and brown for the late phase. **(B)** illustrates with shaded areas of similar colors the three phases identified during the decomposition experiment. Biomass decline is indicated with white squares. Nutrient releases are shown as the remaining percentage of nutrients, %N (in green) and %P (in orange), in *Sargassum* litter. Solid black circles within the squares indicate the timepoints selected for the bacterial community characterizations. Box plots describing the variability in species richness **(C)** and Shannon’s **(D)** and Simpson’s **(E)** diversity indices for each of the clusters/phases identified are also indicated in green (early), red (middle), and brown (late) colors.

**Table 1:** Results of the PERMANOVA and ANOSIM tests to assess for significant differences among the three decomposition phases identified: early, middle, and late.

PERMANOVA	Middle	Late
Early	Adjusted p = 0.003	Adjusted p = 0.012
Late	Adjusted p = 0.003	
ANOSIM	Middle	Late
Early	Adjusted p = 0.003	Adjusted p = 0.018
Late	Adjusted p = 0.006	

At the class level (**Figure 4A**), Proteobacteria (specifically Alphaproteobacteria and Gammaproteobacteria) and Bacteroidota (specifically Bacteroidia) dominated the bacterial community across the three phases. Gammaproteobacteria decreased in relative abundance from the early to the middle phase, while Alphaproteobacteria and Bacteroidia increased (**Figure 4**). Kruskal-Wallis tests (p<0.05) indicate significant differences between phases at both the class and family levels (**Supplementary Figure S5**). Within the Gammaproteobacteria (**Figure 4B**) the early decomposition phase was dominated by *Vibrionaceae*, a family that decreased drastically in abundance after day 3 (Kruskal-Wallis, p<0.05). *Alteromonadaceae* and *Oleiphilaceae* dominated the middle phase, while *Cellvibrionaceae* and *Halomonadaceae* did in the late phase. Within the *Alphaproteobacteria*, the dominant family in all phases was *Rhodobacteraceae* (**Figure 4C**), whereas within Bacteroidia, the dominant families were *Cyclobacteriaceae*, *Flavobacteriaceae*, *Prolixibacteraceae*, and *Saprospiraceae* (**Figure 4D**). *Cyclobacteriaceae*, *Cyclobacteriia*, increased progressively in abundance from early to middle and late phases, whereas *Flavobacteriaceae* and *Prolixibacteriaceae* decreased drastically in the late phase. Notably, *Saprospiraceae*, a family characterized by the presence of species capable of degrading complex organic macromolecules (Mcllroy and Nielsen, 2014), decreased in abundance in the middle phase and increased again in the late phase (**Figure 4D**).

**Figure 4:**
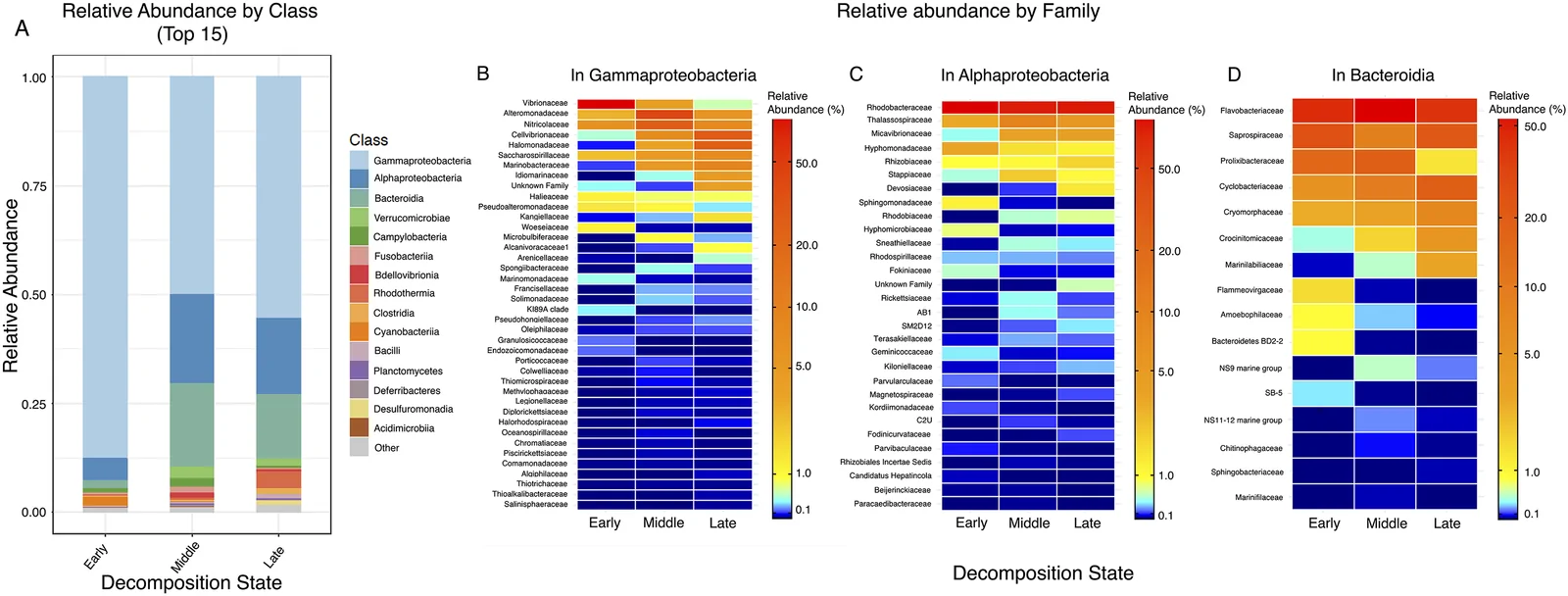
Taxonomic variation of the bacterial microbiome during the decomposition process. **(A)** Variation in relative abundance at the Class level for each phase of the decomposition process; **(B)** at the family level for Gammaproteobacteria; **(C)** for Alphaproteobacteria; and **(D)** for Bacteroidia.

Looking at the families with relatively lower abundance within the Gammaproteobacteria, *Pseudoalteromonadaceae* and *Marinomonadaceae* were more abundant in the early phase; *Spongliibacteraceae*, *Solimonadaceae, Halieaceae, Francisellaceae*, and *Diploricketsiaceae* in the middle phase; and *Pseudohongiellaceae, Marinobacteraceae, Idiomarinaceae, Halorhodospiraceae, Arenicellaceae, Algiphilaceae*, and *Alcanivoracaceae* in the late phase (**Figure 4B**). Additionally, *Thioalkalibacteraceae* was only present in the late phase. For less abundant families within the *Alphaproteobacteria*, NS9 and NS11–12 marine groups, *Crocinitomicaceae* and *Chitinophagaceae* were more abundant in the middle phase, while *Marinilabiliaceae* and *Cryomorphaceae* increased in abundance in the late phase.

### 3.4 Variability in predicted microbial functions

The computational FAPROTAX tool (Functional Annotation of Prokaryotic Taxa; version 1.2.12) (Chen et al., 2025) assigned 522 out of 1428 OTUs (36.5546%) to at least one functional group, predicting a functional profile dominated by chemoheterotrophy and aerobic chemoheterotrophy. The contribution by class and by family to these functional predictions is illustrated, respectively, in **Figures 5** and **6**. Chemoheterotrophy and aerobic chemoheterotrophy activity decreased significantly in the middle and late phases, although they continued to dominate the predicted functional profile. Nitrate reduction and the activity of photosynthetic processes also dominate in the early phase. The importance of these processes decreased in the middle phase, while fermentation, hydrocarbon degradation, and dissimilatory nitrogen reduction processes, including nitrate/nitrite respiration/denitrification, increased in the late phase. The predicted functions and taxa were significantly different between phases (Kruskal-Wallis significance tests; **Supplementary Figure S6**).

**Figure 5:**
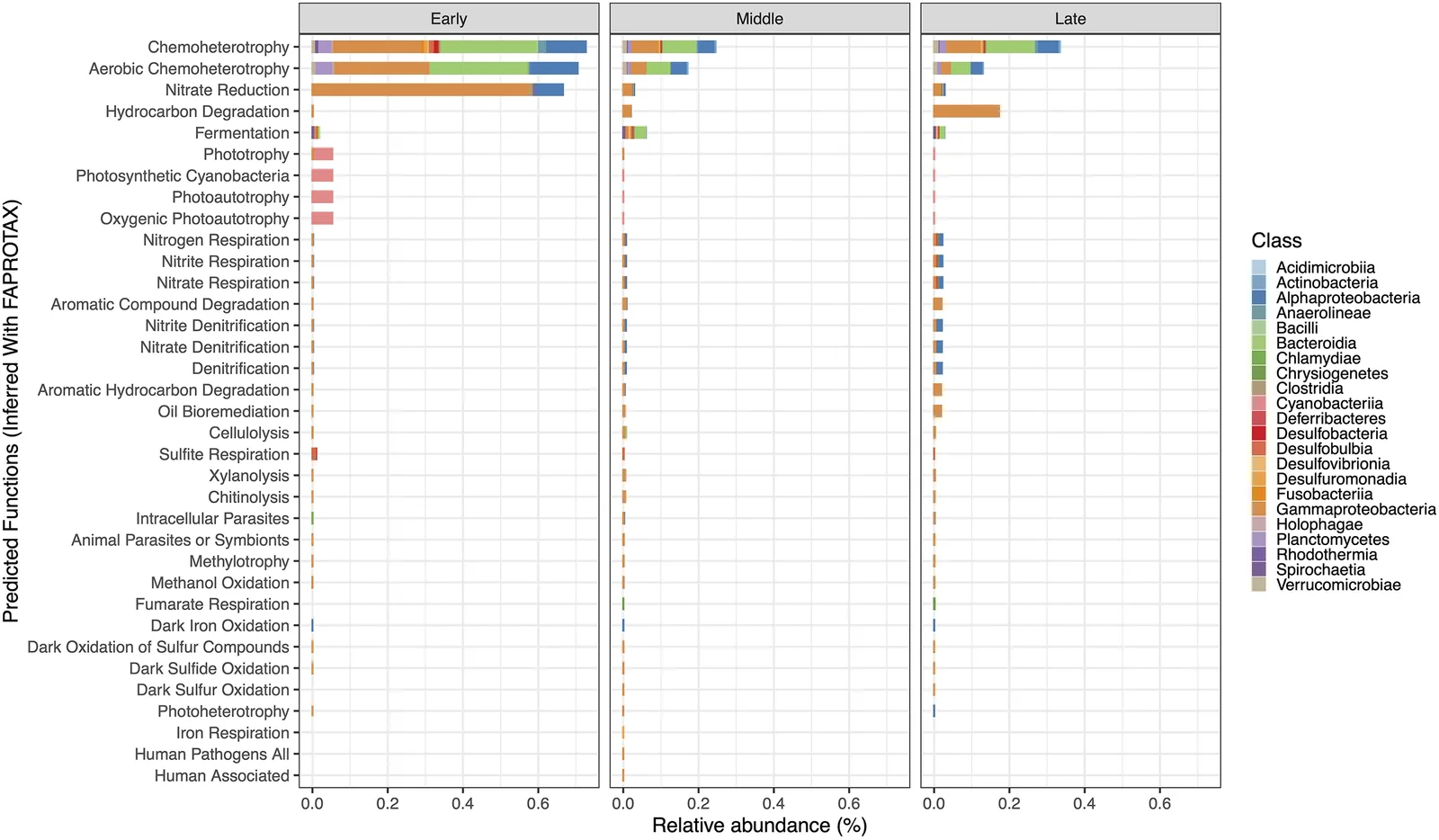
Contribution by Class to the functions predicted by the FAPROTAX computational tool. The analysis allowed recognizing differences between decomposition phases based on Kruskal-Wallis test (p< 0.05; **Supplementary Figure S6**). Significant differences are indicated with asterisks.

**Figure 6:**
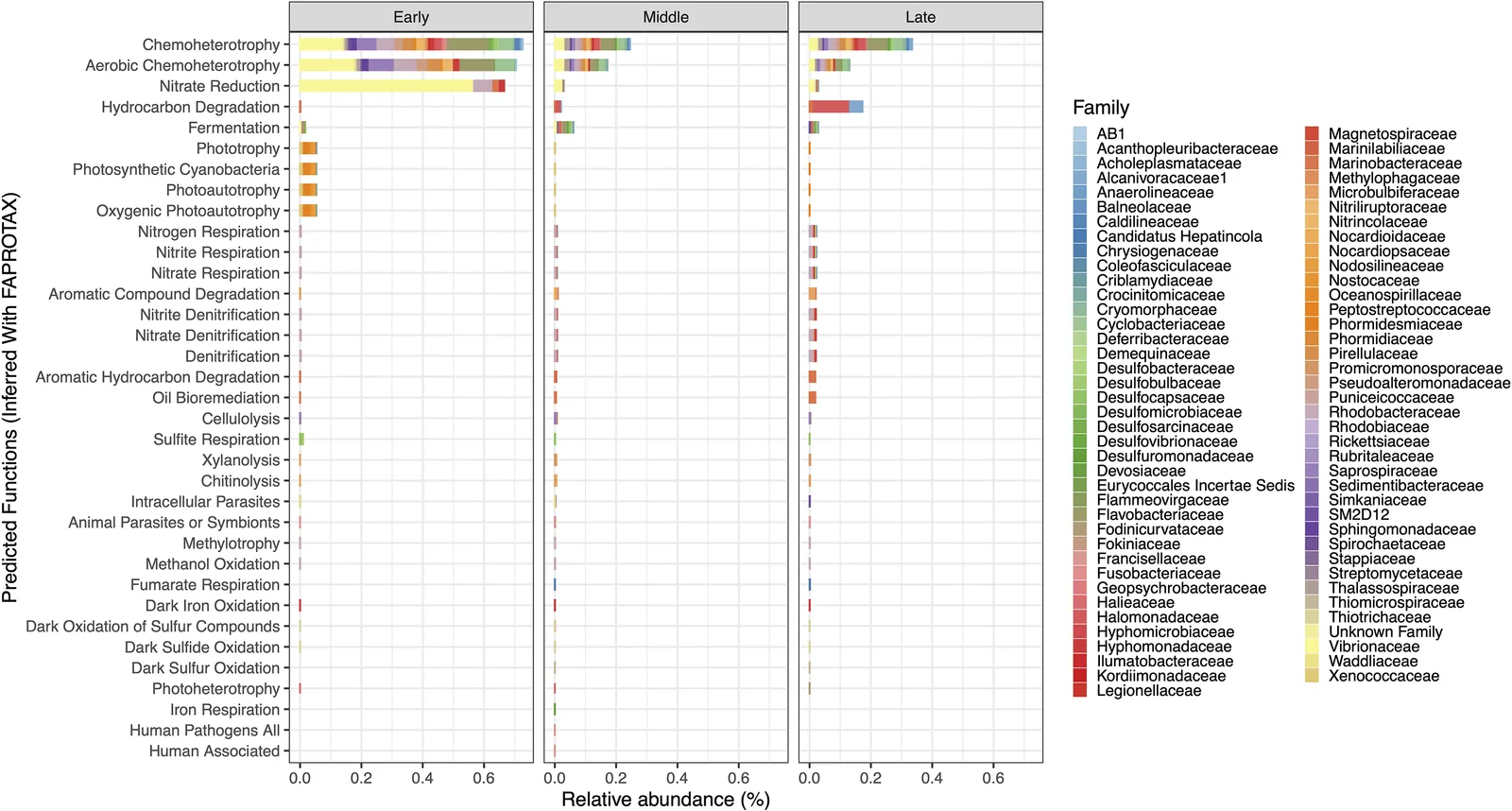
Contribution by Family to the functions predicted by the FAPROTAX computational tool. The analysis allowed recognizing differences between decomposition phases based on Kruskal-Wallis test (p< 0.05; **Supplementary Figure S6**). Significant differences are indicated with asterisks.

On the other hand, PICRUSt2 (Phylogenetic Investigation of Communities by Reconstruction of Unobserved States) inferred among the top 50 most significantly differentially abundant KEGG ortholog (KO) functions across decomposition phases (LinDA with BH correction; adjusted p < 0.05) several functions involved in the biomineralization of nitrogen and phosphorus-containing compounds (**Figure 7**). The middle and late phases of *Sargassum* decomposition were found enriched in nitrous oxide reductase, nitrous oxidase accessory protein, and nitrate reductase (NO-forming). Predicted enzymes involved in amino acid metabolism, including tyrosine ammonia-lyase, taurine-2-oxoglutarate transaminase, and 5-methylthioadenosine/S-adenosylhomocysteine deaminase, also increased consistently from early to middle and late decomposition. Putative aminopeptidase was relatively low in the early phase of decomposition and peaked in the middle phase, while phosphonopyruvate decarboxylase increased across decomposition phases, peaking in late decomposition (**Figure 7**). PICRUSt2 also predicted statistically significant increases in 17 features associated with phosphonate metabolism and transportation (LinDA with BH correction; adjusted p < 0.05). Some of them peaked in the middle phase, while others did in the late phase (**Figure 8**). Remarkably, two features associated with phosphonate activity, 2-aminoethylphosphonate-pyruvate transaminase and a substrate-binding protein of the phosphonate transport system, were higher in the early phase and decreased to middle and late decomposition (**Figure 8**).

**Figure 7:**
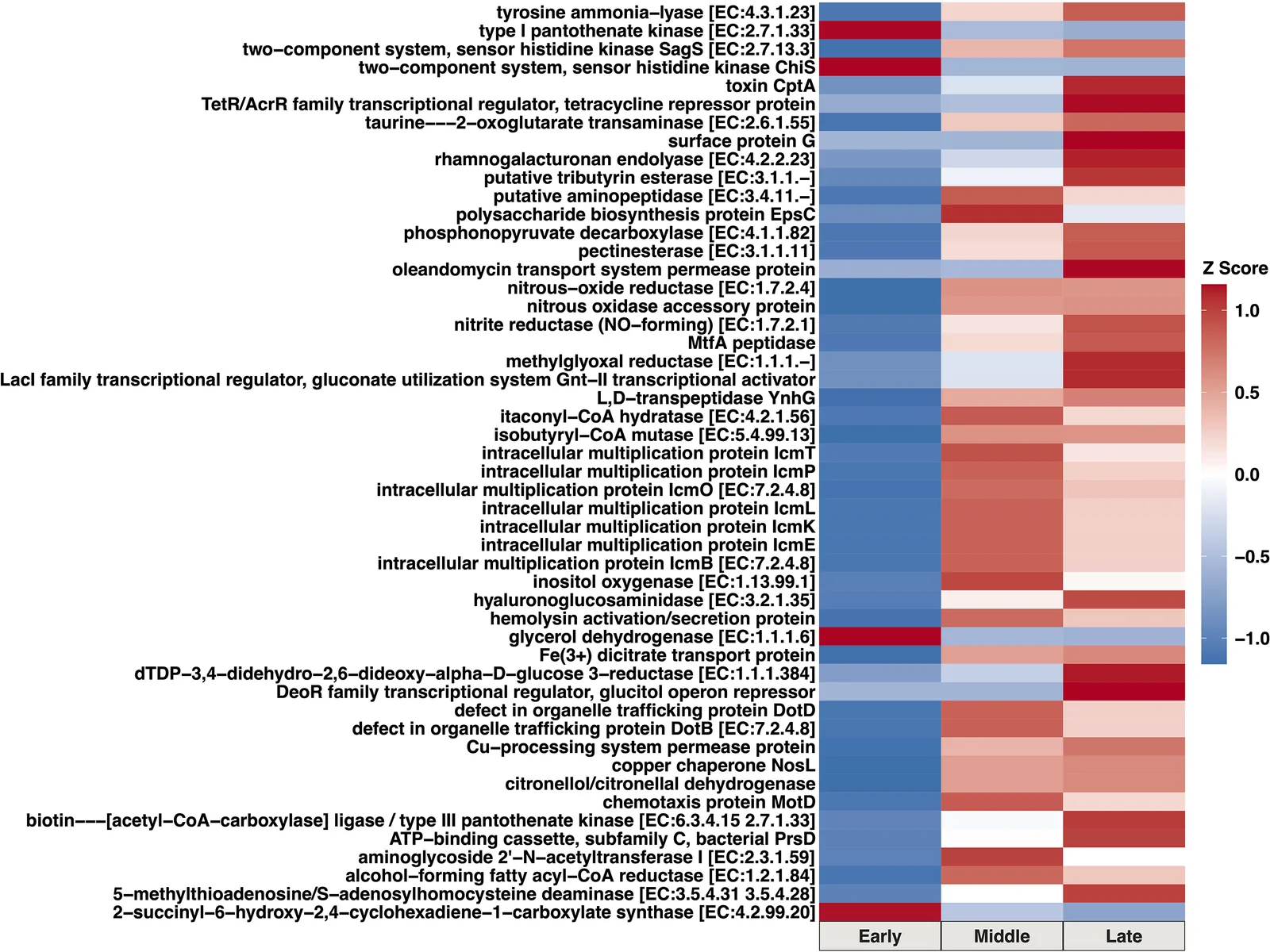
Variation in PiCRUSt2 inferred functions during the decomposition process. Only the top 50 functions showing the most significant variations identified using LinDA with Benjamini-Hochberg (BH) FDR correction (adjusted p < 0.05) are shown.

**Figure 8:**
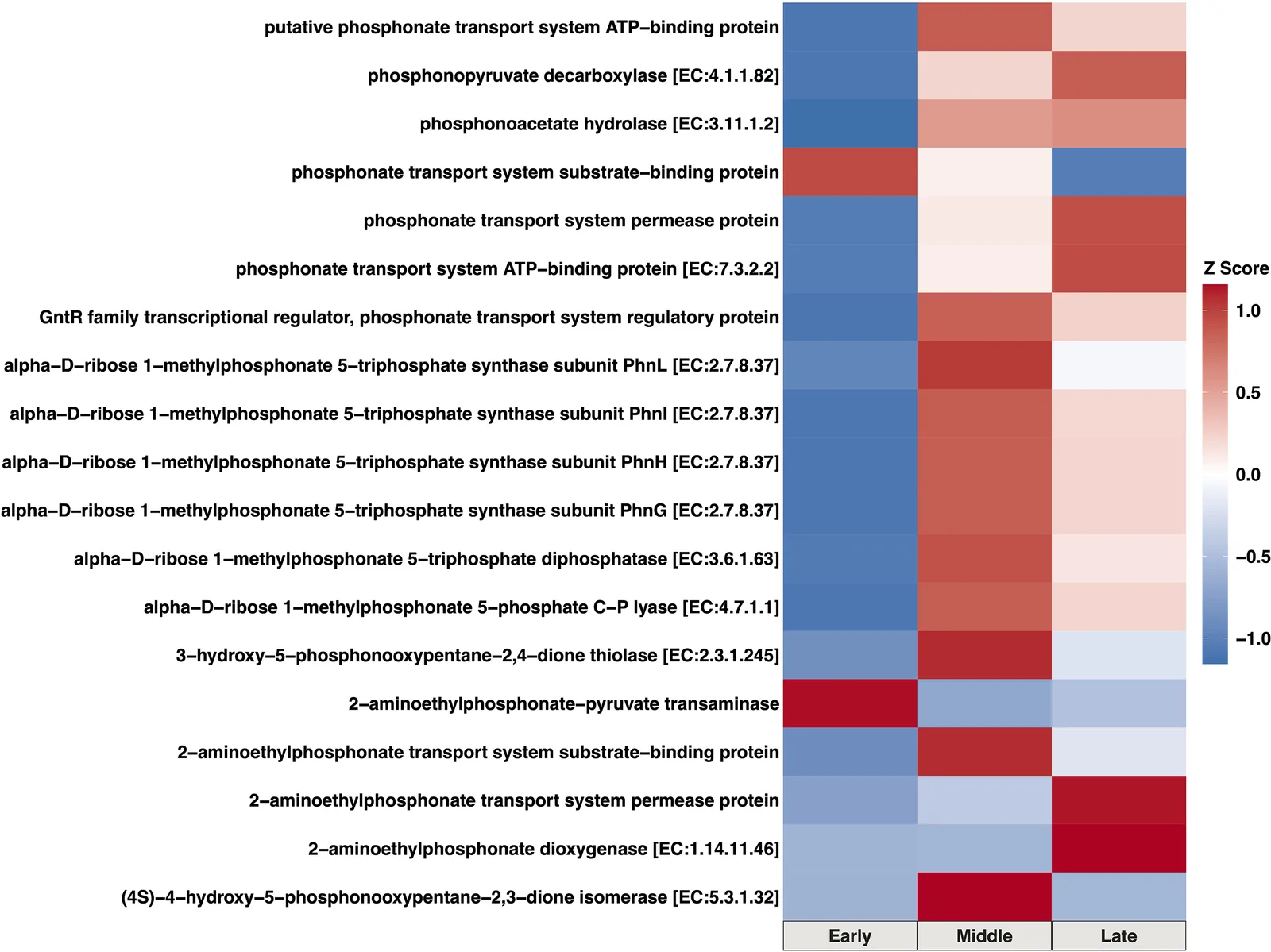
Variation in PICRUSt2 inferred functions related to organophosphonate degradation during the decomposition process. Only functions showing significant variations identified using LinDA with Benjamini-Hochberg (BH) FDR correction (adjusted p < 0.05) are shown.

## 4 Discussion

### 4.1 Sargassum traits and biomass dynamics

This functional analysis of pelagic *Sargassum* confirmed the enormous source of organic carbon and nutrients that the highly productive ecosystem established since 2011 in the North Equatorial Recirculation Region (NERR) currently represents for the coastal ecosystems of this region and, particularly, for Caribbean coral reefs. The estimated *Sargassum* growth rates were, after correcting for biomass losses (gRGR), within the range reported in the literature (see Velázquez-Ochoa et al., 2026) although below the rates documented for invasive/opportunistic species (Stiger-Pouvreau et al., 2023) and for *S. fluitans* var. *fluitans* (former *S. fluitans* III) under nutrient-enriched conditions (≈77–109 mg gFW^-^¹ d^-^¹) (Hanisak and Samuel, 1987; Leemans et al., 2025; Velázquez-Ochoa et al., 2026). The significant interference of biomass loss found in the growth determinations offers an explanation for the large variability between replicates and the frequent negative growth values commonly reported in the literature for the estimations of pelagic *Sargassum* growth, even for control samples under favorable experimental conditions (Velázquez-Ochoa et al., 2026). The lack of reliability of the values estimated in our second experiment exemplifies this problem. Remarkably, using a more homogeneous population of initial samples with similar age and photoacclimation condition and reducing self-shading during the incubations, allows for minimizing this experimental problem, leading to more reliable estimates of new growth and biomass loss for *Sargassum*. Hence, ignoring the unreliable results of the second experiment, our study supports the notion that both elevated temperature and nutrient limitation increase *Sargassum* biomass loss rates, which may explain the reported reductions in *Sargassum* growth at 28 °C (Magaña-Gallegos et al., 2023) despite the absence of thermal stress at that temperature (Velázquez-Ochoa et al., 2026).

In contrast with growth estimates, the decomposition rates and half-life values found for *Sargassum* detritus uncovered a distinctive algal trait with values more common for phytoplankton and freshwater plant litter than for marine algae, which range between -0.001 and -0.03 d^-^¹ for decomposition rates and between 25 and 700 days for detritus half-life (Enríquez et al., 1993). Such a rapid decomposition may help explain the fast O_2_ depletion observed during massive beaching events and the resulting dominance of anaerobic processes with the consequent emission of toxic gases. The public health problems generated at the coast have received significant attention, but unfortunately, they have also overshadowed the main ecological threat that the massive *Sargassum* presence in the Atlantic poses to coastal ecosystems: their rapid eutrophication (Velázquez-Ochoa and Enríquez, 2023).

### 4.2 Microbial community succession during decomposition and nutrient release

A bacterial community dominated by Alphaproteobacteria, Gammaproteobacteria, and Bacteroidia, similar to other previous descriptions for *Sargassum* (Hervé et al., 2021; Mendonça et al., 2024) and macroalgae (Hu et al., 2023), may be responsible for such rapid algal decomposition. Functional inference analyses suggest a succession pattern similar to other processes described for terrestrial ecosystems (Louca et al., 2016; Hu et al., 2025), unveiling three distinct phases, early, middle, and late, with greater species richness in the middle and late phases compared to the early stages. Predicted functional profiles by FAPROTAX were dominated by chemoheterotrophy and aerobic chemoheterotrophy across all decomposition stages. It should be noted, however, that the results of this study may be biased towards gram-negative bacteria, as we may have failed to capture the full diversity of bacterial taxa present in the community.

The community changes predicted coincided with highly contrasting nutrient release rates from *Sargassum* detritus: very rapid, particularly for phosphorus, in the early and late phases and significantly slower in the middle phase. Two groups known to be co-limited by inorganic nitrogen and phosphorus—Bacteroidia and *Roseobacter* (*Rhodobacteraceae*) (Sebastián and Gasol, 2013)—were predicted to increase in relative abundance in the middle phase, coinciding with a significant slowdown in detritus nutrient release. Their abundance remained relatively constant in the late phase, during which a renewed rapid release of phosphorus was observed. We interpreted these findings considering that the reduced accessibility to organic nutrients for the bacterial community that initiated the mineralization process may have favored the success of groups more resistant to nutrient limitation in the middle phase, leading to the diversification of the microbial community observed in this phase, which may reflect the need to exploit a more diverse and complex array of organic compounds in *Sargassum* detritus. The succession pattern for nitrogen mineralization predicted by PICRUSt2 (Phylogenetic Investigation of Communities by Reconstruction of Unobserved States) suggests that the breakdown of *Sargassum* biomass into organic nitrogen compounds was facilitated by the bacterial community established during the intermediate-middle phase of decomposition. Subsequently, during the late phase and associated with the decrease in *Sargassum* biomass, the analysis predicts that protein and peptide metabolism slows down, while organic nitrogen metabolism increases to more likely facilitate the biomineralization—into inorganic nitrogen—of the released organic nitrogen compounds. Therefore, these results are consistent with the established idea that nutrients determine the decomposition rates of plant/algal litter (Enríquez et al., 1993) and support the conclusion that nutrients also drive specific patterns in microbial community succession during marine debris decomposition, as it has already been shown for terrestrial plant litter (Chen et al., 2025).

### 4.3 Microbial community in the early phase: diazotrophs.

The functional predictions of photosynthesis, phototrophy/photoautotrophy, and nitrogen respiration found for the early phase support the well-documented presence of epiphytic diazotrophs (N_2_ fixation organisms), diazotrophic activity, and *Cyanobacteriota* on the surface of pelagic *Sargassum* (i.e., Cox et al., 2023; Léger-Pigout et al., 2024). This diazotrophic activity has also been supported by direct measurements of epiphytic N_2_ fixation on pelagic *Sargassum* (i.e., Johnson et al., 2023); the identification/quantification of the gene encoding a subunit (nfH gene) of the nitrogenase involved in N_2_ fixation (Léger-Pigout et al., 2024); and the isolation of a heterocyst-bearing blue-green alga, *Dichothrix fucicola* (Carpenter, 1972), the filamentous cyanobacteria *Calothrix* (Calotrichaceae, Nostocales) together with one LPP-type (Phlips et al., 1986), and nine cyanobacteria belonging to the genus *Oscillatoria* (Phlips and Zeman, 1990). Therefore, the detection of *Cyanobacteriia* in the early phase of the decomposition experiment of this study indicates that we may have captured in the analysis the original microbiome of *Sargassum*, a plausible conclusion since the decomposition experiment was initiated with fresh, living samples rather than detritus. This interpretation is also supported by the fact that classes such as *Actinomycetota*/*Actinobacteria* and *Cyanobacteriota*/*Cyanobacteriia*, which are typically more abundant in floating populations and less common in stranded *Sargassum* (Hervé et al., 2021), were also identified, with *Cyanobacteriia* showing a decreasing abundance from early to late phases. Diazotroph abundance decreases in nitrogen-enriched coastal waters in favor of denitrifiers, an observation associated with increases in *Sargassum* (δ^15^N) (Léger-Pigout et al., 2024). Populations from the Gulf of Mexico associated with wastewater discharges also show higher (δ^15^N) values (Rooker et al., 2006; McGillicuddy et al., 2023), while those in the Sargasso Sea, central tropical Atlantic, Caribbean, and Gulf Stream often exhibit low (δ^15^N) (Baker et al., 2018; Lapointe et al., 2021; Vázquez-Delfín et al., 2021; McGillicuddy et al., 2023; Velázquez-Ochoa and Enríquez, 2023; Hu et al., 2025) supporting the conclusion that the atmosphere is the main nitrogen source for pelagic *Sargassum* under nitrogen-limiting conditions acquired through N_2_ fixation by diazotrophic organisms or atmospheric deposition (Mendonça et al., 2007). This parameter, (δ^15^N), also varies with temperature (Lapointe et al., 2021), thallus age (Phlips and Zeman, 1990), within *Sargassum* rafts, and between the genetic variants, with *S. fluitans* var. *fluitans* the species with the highest (δ^15^N) values (Changeux et al., 2023). Consistent with this, the already low (δ^15^N) values reported by Vázquez-Delfín et al. (2021) for populations arriving in the Mexican Caribbean decrease as the relative abundance of *S. natans* var. *natans* (former *S. natans* I) increases and *S. fluitans* var. *fluitans* decreases—both abundances are highly correlated (r = -0.92; p < 0.01; see **Supplementary Figure S8**)—supporting more efficient diazotrophic activity in the microbiome of *S. natans* var. *natans* compared to that of *S. fluitans* var. *fluitans*. This finding also aligns with the conclusion that *S. natans* var. *natans* is the genetic variant more resilient to starvation and thus better adapted to survive under oligotrophic oceanic conditions (Velázquez-Ochoa et al., 2026). The information available to date gives support to a key role of mutualistic associations between *Sargassum* and its microbiome to enhance algal survival under nutrient-limiting conditions, an area that has not yet been sufficiently explored. Diazotrophs allow enhancing nitrogen bioavailability for the algal host while being sustained by *Sargassum* organic exudates. Offshore populations of pelagic *Sargassum* in the NERR or Sargasso Sea host higher abundances of diazotrophs compared to coastal samples or benthic *Sargassum* species (Léger-Pigout et al., 2024), further supporting the beneficial role of these mutualistic associations in enhancing algal performance under nitrogen limitation. Diazotrophs typically constitute about 2% of the bacterial community in offshore *Sargassum* populations (Léger-Pigout et al., 2024), with non-cyanobacterial diazotrophs being predominant (≈76%), underlining the interest that this group deserves in further research.

### 4.4 Sargassum access to the organic phosphorus pool.

While diazotrophic activity in the *Sargassum* microbiome has been widely documented (Parr, 1939; Venter et al., 2004; Rooker et al., 2006; Mendonça et al., 2007; White and Metcalf, 2007; Sosa et al., 2017; Léger-Pigout et al., 2024; Cox et al., 2025), there is not yet direct evidence implicating *Sargassum*, its microbiome, or their combination in the increased rates of extracellular phosphatase activity observed in the Sargasso Sea compared to the continental shelf (Hervé et al., 2021) or in the enhanced phosphonate degradation activity (Cox et al., 2023) associated with higher relative abundance of bacterial groups such as *Rhodobacteraceae* (Cox et al., 2025). *Rhodobacteraceae* are capable of degrading organo-phosphonates via the carbon-phosphorus lyase (C-P lyase) pathway (Venter et al., 2004; White and Metcalf, 2007), a multi-enzyme complex that cleaves the stable C-P bond in phosphonates, releasing inorganic phosphate (orthophosphate) for cellular use. This canonical pathway (White and Metcalf, 2007) is present in many groups within the *Sargassum* microbiome (Hu et al., 2023; Cox et al., 2025). Both the presence of phosphonate activity and the ability to release extracellular phosphatases (APases, acid or alkaline) can significantly enhance algal access to the external P_org_ pool. In our analysis, *Rhodobacteraceae* was predicted as the dominant family of Alphaproteobacteria across all three phases, with increased relative abundance in the middle and late phases. Remarkably, PICRUSt2 predicted significant increases in 17 features associated with phosphonate metabolism and transportation (LinDA with BH correction; adjusted p < 0.05). Most of them peaked in the middle and late phases of the decomposition process, highlighting a potential contribution of phosphonate metabolism during *Sargassum* mineralization.

### 4.5 Potential role of the Sargassum microbiome in rapid thallus mineralization and carbon/nutrient fluxes

We measured in this study biomass loss rates for young segments of *Sargassum* between 9 mg g DW d^-1^ (26°C) and 19 mg g DW d^-1^ (30°C), suggesting the presence of a bacterial community of decomposers in the *Sargassum* microbiome, capable of accelerating thallus turnover rates at higher temperatures, reducing thallus life span. These values were estimated under no nutrient limitation, as the values for *Sargassum* biomass loss under starvation were, unfortunately, not reliable. However, samples exposed to nutrient limitation in the first experiment presented similar thallus elongation rates and half-node production to samples exposed to sufficient nutrient availability during the incubation, although at the cost of greater biomass loss and lower life span. Understanding the environmental conditions that favor biomass loss is, then, essential for investigating the spatial and temporal variability of *Sargassum* in the Atlantic, the subject of intensive monitoring using remote sensing tools (Wang et al., 2019; Jouanno et al., 2023). Our analysis allows us to conclude that biomass loss rates increase under starvation and higher temperatures, giving support to the large biomass declines of *Sargassum* reported in late summer to early autumn (Wang et al., 2019; Jouanno et al., 2023) right after the maximum summer temperature in August (Scheufen et al., 2017). They also support the prediction of significant declines in the biomass of the floating population as *Sargassum* moves away from nutrient sources and faces starvation on its journey across the Atlantic and the Caribbean. Larger biomass loss rates are also expected in older segments and under suboptimal physiological conditions. Hence, further research is needed to fully understand how biomass loss varies under different nutrient, light, and temperature regimes. Likewise, current growth estimates for pelagic *Sargassum* need to be re-evaluated, taking into account variations in biomass loss and thallus turnover rates. This information is fundamental for explaining the seasonal and interannual variability of pelagic *Sargassum* in the central Atlantic using predictive models.

The presence in the microbiome of pelagic *Sargassum* of an epiphytic bacterial community capable of fixing N_2_ and recycling organic nutrients—particularly phosphorus—under nutrient-limiting conditions could also explain *Sargassum*’s robustness to starvation and its great ecological success in colonizing the oligotrophic ocean surface. In this respect, two features associated with phosphonate activity, 2-aminoethylphosphonate-pyruvate transaminase and a substrate-binding protein of the phosphonate transport system, were predicted in this analysis for the early phase and decreased to middle and late decomposition.

The identification of these microbial groups was beyond the scope of the present study, but we provide here insight on the bacterial taxa that may be preferentially involved, particularly those able to accelerate organic phosphorus mineralization. Among Bacteroidia, dominant families with potential roles in rapid detritus mineralization and phosphorus release include *Flavobacteriaceae*, key in polysaccharide degradation (Hu et al., 2023); *Saprospiraceae*, known for breaking down complex organic compounds (Mcllroy and Nielsen, 2014); and C*yclobacteriaceae*, with members capable of degrading polysacharides and lipids (Pinnaka and Tanuku, 2014). Prominent groups recognized as core members of the *Sargassum* microbiome (Cox et al., 2025), including *Vibrionales, Rhodobacterales, Alteromonadales*, *Pseudomonadales*, and *Oceanospirillales*, as well as *Cyanobacteriia* and *Planctomycetota*, warrant particular attention, especially the dominant families *Pseudomonadota, Vibrionaceae, Alteromonadacea*, and *Rhodobacteraceae*. Metagenomic and qPCR data suggest that *Vibrio* may participate in nitrogen fixation and organic phosphorus utilization, highlighting its potential to establish beneficial associations with the pelagic alga and/or play a beneficial role in biogeochemical cycling (Sosa et al., 2017; Cox et al., 2025). The two features associated with phosphonate activity predicted by PICRUSt2 in the early phase and that were progressively decreased to the middle and late phases may also reflect some epiphytic activity that should be explored in future research. The microbial groups responsible for accelerating *Sargassum* mineralization may also participate in explaining the ecological success of pelagic *Sargassum* on the oceanic surface and the current nutrient enrichment of the Caribbean (Baker et al., 2018; Wang et al., 2019; Jouanno et al., 2023; Jung et al., 2025), helping elucidate the mechanisms that govern macroalgal responses to starvation and how frequently these mutualistic associations occur in natural populations. Our study, then, highlights the essential role that microbial communities may play in enhancing algal resilience to starvation by the establishment of mutualistic associations with their hosts. while emphasizing their pivotal role in mediating carbon and nutrient fluxes within global biogeochemical cycles.

### 4.6 Implications for the oceanic phosphorus cycle

On geologic timescales, phosphorus is considered a key limiting nutrient of marine primary production and blue carbon storage, playing a central role in atmospheric CO_2_ regulation. Several studies have argued that changes in the oceanic phosphorus cycle have impacted climate, triggering shifts on glacial-interglacial timescales (Ruttenberg, 2014). Preferential phosphorus mineralization for the oceanic dissolved organic matter (DOM) has also been documented as a general trend, with the North Atlantic region exhibiting larger anomalies with respect to the Redfield stoichiometry (Letscher and Moore, 2015). This finding supports the idea that biogeochemical cycles in this region are strongly P-depleted, particularly in the Sargasso Sea. Our study provides new insight into the biological processes that may explain these observations and inferences (Letscher and Moore, 2015), emphasizing the utility of a more profound understanding of the bacterial communities and biological processes responsible for organic phosphorus mineralization and thereby able to influence oceanic biochemical cycles, atmospheric carbon fluxes, and, ultimately, changes in global climate. A better mechanistic knowledge of these biological processes will also facilitate the development of quantitative models for predicting biogeochemical carbon and nutrient fluxes in the ocean and coastal ecosystems, as well as new technologies capable of mitigating the environmental consequences of the massive accumulation of algal waste on the coast.

## 5 Conclusions

This functional analysis of pelagic *Sargassum* biomass dynamics confirms the severe threat of eutrophication that the highly productive ecosystem established in the North Equatorial Recirculation Region (NERR) currently represents for coastal environments and marine health in the region and, particularly, for Caribbean coral reefs. Although growth estimates were below the values expected for opportunistic/invasive species, the exceptional trait found for its mineralization rates is a call of attention for the vast flux of nutrients that is currently transported from the Atlantic to the Caribbean and rapidly released when mass algal tides arrive on the coast and beach, inducing a fast depletion of oxygen and toxic gas emissions with known consequences for public safety. This *Sargassum* trait may also be responsible for a rapid thallus turnover rate that can explain larger biomass loss under starvation and elevated temperature together with the shortcomings previously identified for the published literature’s growth estimates. These findings highlight the putative presence on the *Sargassum* surface of a microbial community capable of an efficient recycling of organic nutrients that should be investigated. The well-known presence of diazotrophs in this community, along with the possible presence of groups capable of rapid phosphorus mineralization, strengthens the interest in elucidating the microbiome’s contribution to the ecological success of *Sargassum* in the oligotrophic oceanic surface, as well as the evolutionary role that these mutualistic associations may play in enhancing algal resilience to starvation.

## Data Availability

The datasets presented in this study can be found in online repositories. The names of the repository/repositories and accession number(s) can be found below: https://www.ncbi.nlm.nih.gov/, https://www.ncbi.nlm.nih.gov/bioproject/PRJNA1419322.

## Supplementary material

Supplementary Figure 1 Experimental set-up used in the three growth experiments performed.

Supplementary Figure 2 Comparison of the contrasting biomass used for the initial samples between growth experiments.

Supplementary Figure 3 Experimental set-up used in the decomposition experiment.

Supplementary Figure 4 Comparison of fresh vs. dry weight in pelagic *Sargassum*.

Supplementary Figure 5 Variation in relative abundance of microbiome members of the three bacterial communities of Sargassum detritus decomposers identified (early, middle, and late) and Kruskal-Wallis tests

Supplementary Figure 6 Variation in relative abundance of the predicted functional profiles for the three phases identified in the decomposition process (early, middle, and late) and Kruskal-Wallis tests.

Supplementary Figure 7 Comparison of data for the isotopic signature of nitrogen (δ15N) of Sargassum thalli documented by Vázquez-Delfín et al. (2021).

Supplementary Table 1 Values and the studies that have documented the impact of *Sargassum* tides on oxygen dissolved in coastal waters.

Supplementary Table 2 Values and the studies that have documented the variation in nutrient concentration in coastal waters associated with mass *Sargassum* tides.

Supplementary Table 3 Values and the studies that have documented toxic gas emissions associated with mass *Sargassum* tides.
