# Supplementary material for: Microbial community dynamics mediate rapid biomass loss and phosphorus recycling in pelagic Sargassum
Source: Front Plant Sci. 2026 Oct 5;17:1918885. doi: 10.3389/fpls.2026.1918885 (PMC13679642; doi:10.3389/fpls.2026.1918885)
Supplement: Supplementary file 1 [file DataSheet1.pdf]

Supplementary Material

1 Supplementary Figures

Supplementary Figure S1. Experimental set-up used in the three growth experiments performed. Four tanks per treatment and four replicates per tank (n=16) were used to determine the absolute and relative growth rates of *Sargassum fluitans* III under two nutrient treatments (experiments 1 and 2). In the temperature experiment (experiment 3), three tanks and five replicates per tank were used to determine the growth rates of *S. fluitans* III. Samples were bubbled with oxygen during the incubation, and neutral meshes allowed 20% reductions in solar illumination. Light was monitored during the incubation, and a constant temperature of ≈ 30ºC was maintained. The figure also shows close-ups of the small samples used in experiments 1 and 3.

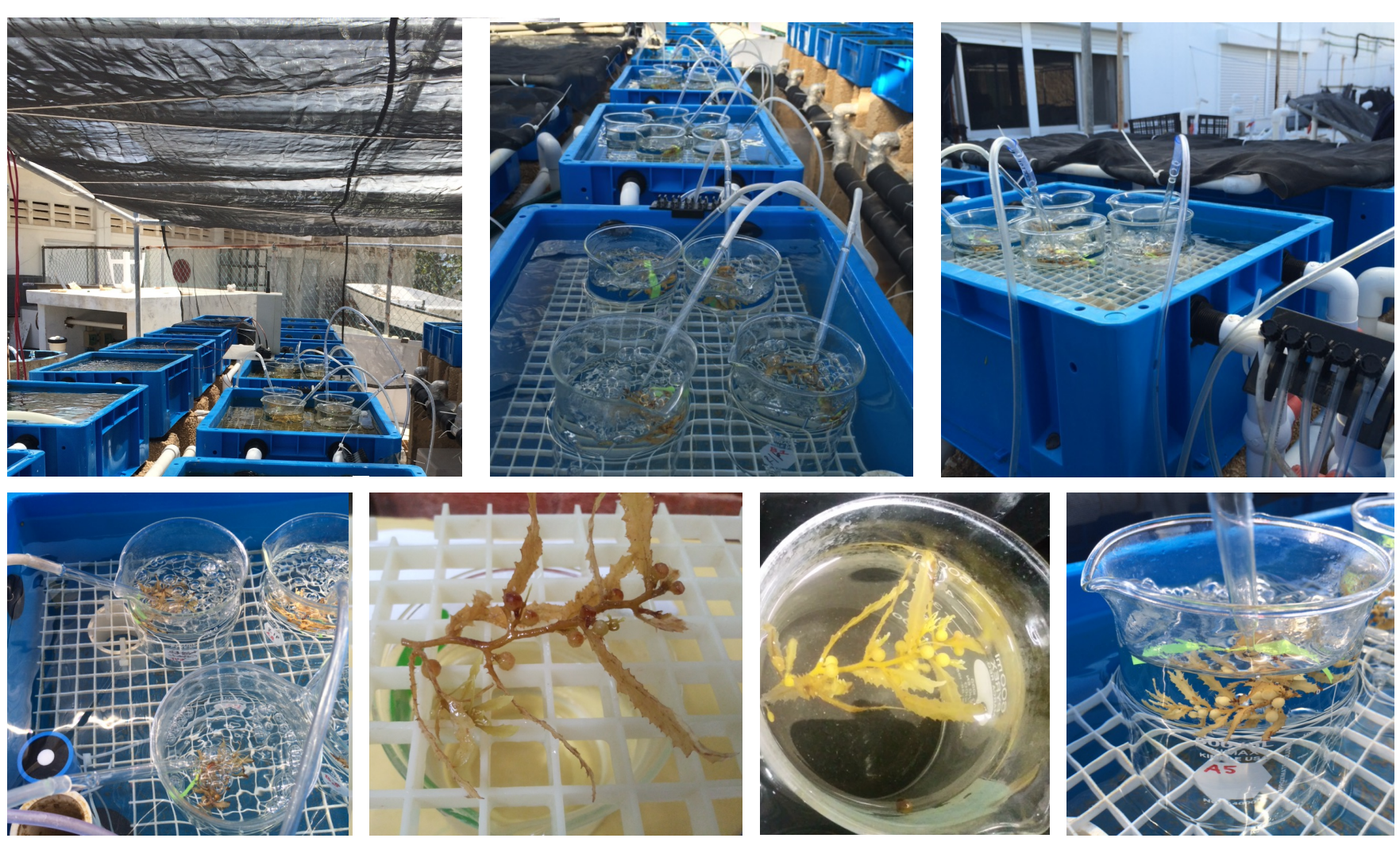

Supplementary Figure S2. Comparison of the contrasting biomass used for the initial samples between the 1 and 3 experiments (images in A and B) and the second experiment (images C and D). In experiments 1 and 3, samples had a fresh weight of 0.44 g FW ± 0.07 and 0.55 g FW ± 0.06, respectively, whereas in experiment 2, the initial fresh weight was one order of magnitude greater (4.8 g FW ±0.7; images C and D in Figure S2). The use of young thalli and a smaller sample size was crucial for having better control of sample illumination during the incubation, minimizing self-shading, and working with a more homogeneous experimental population as the photoacclimatory condition and thallus age of the initial samples were more similar among replicates.

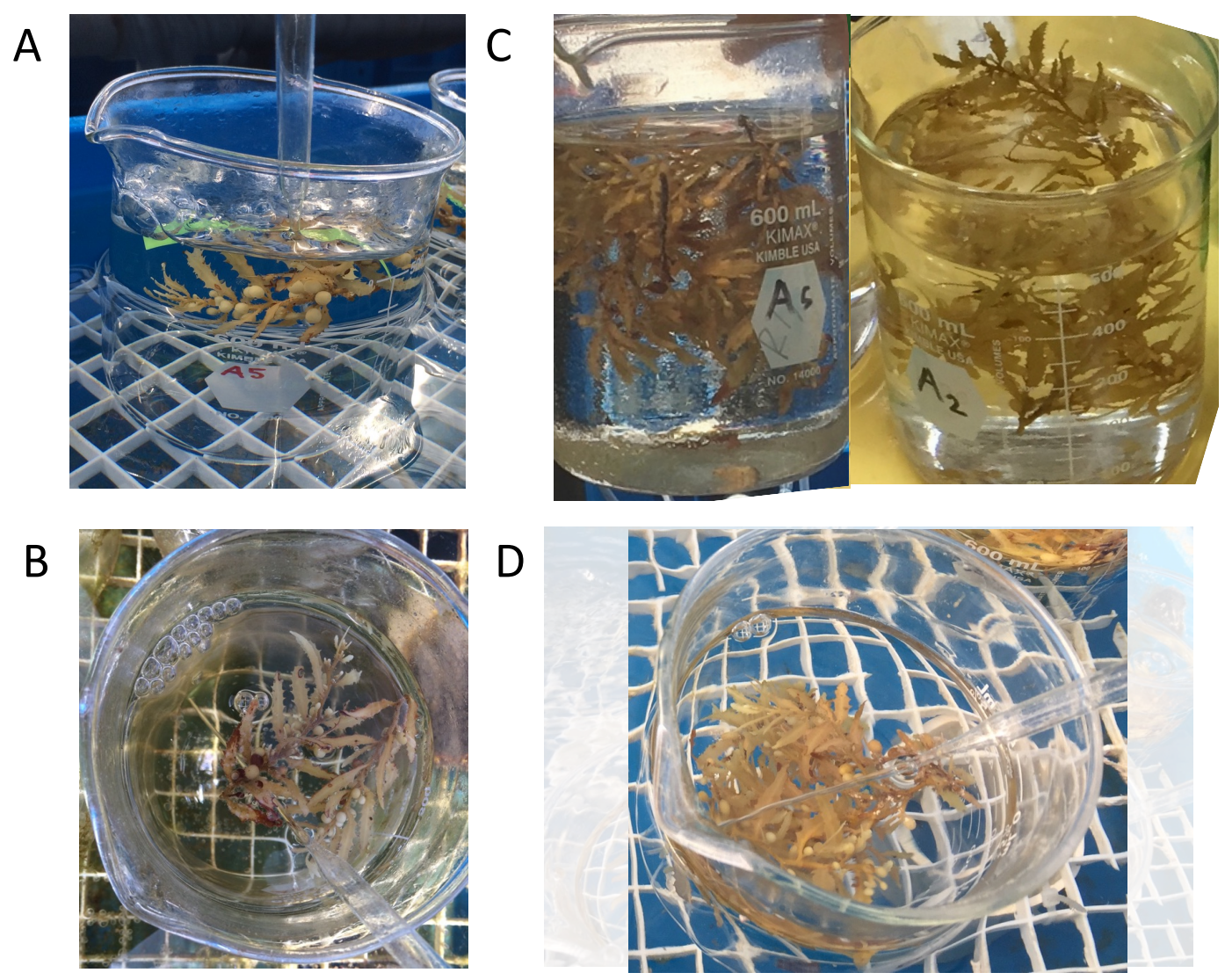

Supplementary Figure S3. Experimental set-up used in the decomposition experiment. Fresh samples of *Sargassum* with their characteristic yellow color were collected in the reef lagoon of Puerto Morelos (Mexican Caribbean), far from the shore, and placed in the experimental tank. Dead material with the first change in color (i.e., reddish brown) was collected at the shore, as indicated by the white circle in image E of Figure S3, and placed at the head of the tank without direct contact with the living sample collected in the ocean, as illustrated in the images S3C and S3D. This dead material was used as "seed biomass” to help in spreading the microbiome of decomposers within the fresh living samples (in yellow color in the images S3C and S3D). Plots S3A and S3B illustrate two initial attempts with pre-weighted “clean” samples, either introduced in closed mesh bags (image S3A) or placed on the top of a tray also made of mesh fabric (image S3B) to monitor detritus weight loss, which failed to initiate the decomposition process. Using dead material as "seed biomass” helped in spreading the microbiome of decomposers within the fresh living samples. Plots S3C and S3D illustrate the third and successful experiment that allowed the estimation of *Sargassum* decomposition rates. Fresh living samples with their characteristic yellow color, as indicated in the image S3E with a dark cycle, were collected directly from the ocean. Dead material with the first change in color (i.e., reddish brown) was collected at the shore, as indicated by the white circle. Both samples were placed in the tank, not in direct contact. The dead material was located at the head of the tank, as illustrated in pictures S3C and S3D. Fresh living *Sargassum* was pre-weighted and introduced in ten cylinders opened on both sides, which were weighted at specific times. Cylinders were replaced with new pre-weighted material from the surrounding biomass after each weighting. In the images integrated in plot F is illustrated the changes of *Sargassum* detritus throughout the experiment.

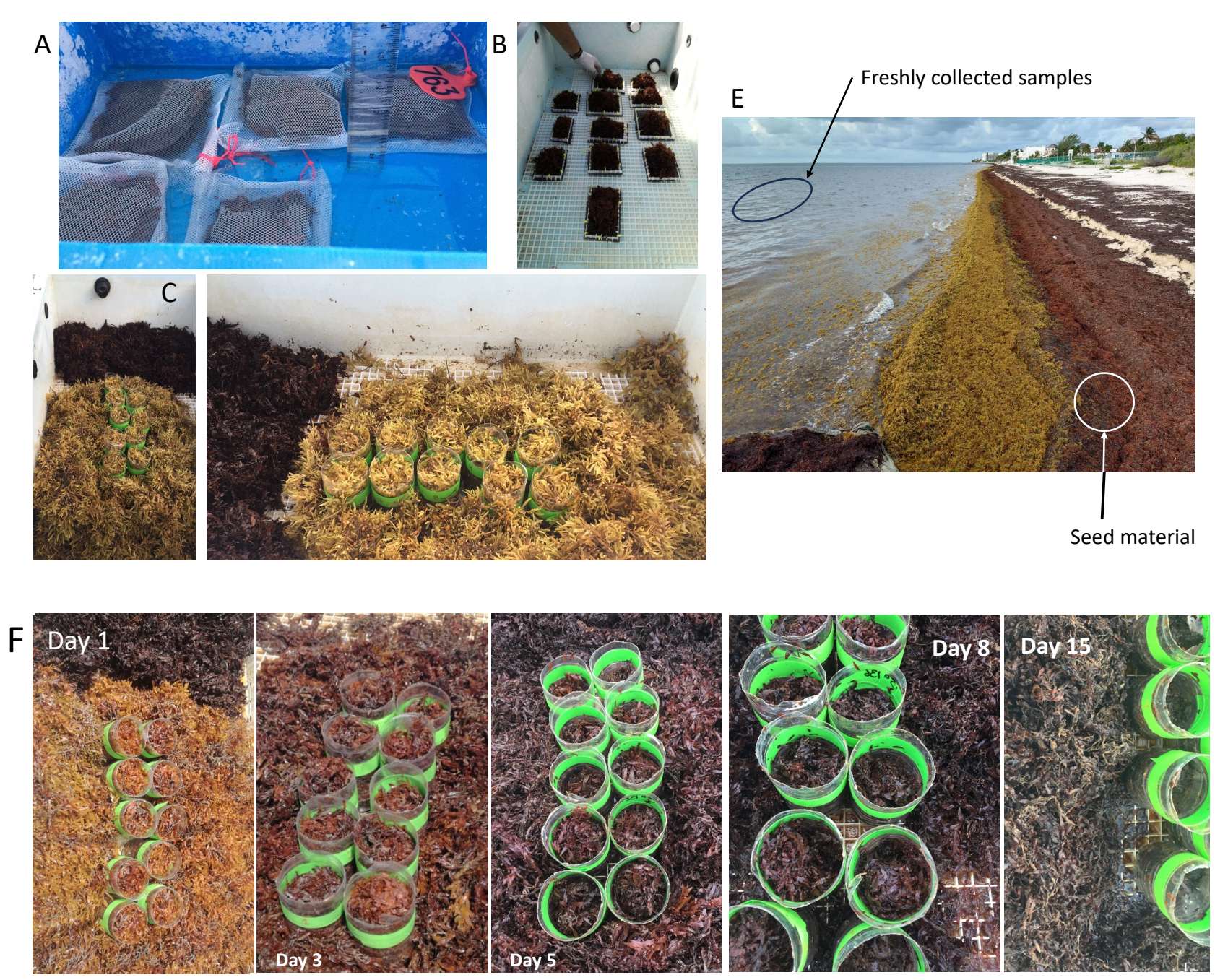

Supplementary Figure S4. Comparison of fresh vs. dry weight in pelagic *Sargassum* (n = 31) (R^2 = 0.93; $p < 0.001$). This association of variation was described by the following equation:

$$\text{Fresh weight} = 0.2 \pm 0.0023 \times \text{Dry weight}$$

and allowed the conversion between both parameters.

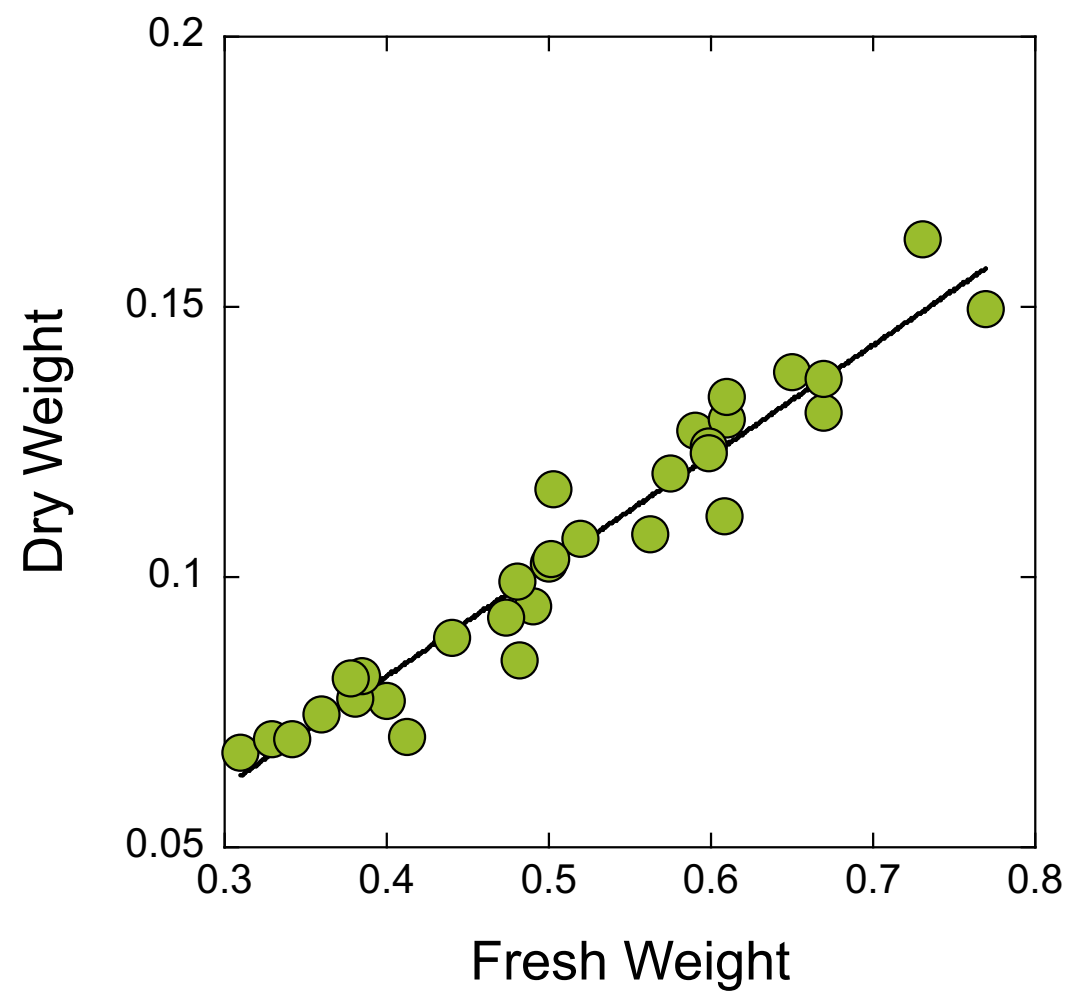

Supplementary Figure S5. Variation in relative abundance of microbiome members of the three bacterial communities of *Sargassum* detritus decomposers identified (early, middle, and late) and Kruskal-Wallis tests: (A) at the Class level; (B) at the Family level for Gammaproteobacteria; (C) at the Family level for Alphaproteobacteria; and (D) at the Family level for Bacteroidia. Dot plots include only taxa that are significantly different based on Kruskal-Wallis tests ($p<0.05$). Dots represent medians and lines represent 95% confidence intervals. Bar graphs illustrate relative abundance for all taxa that are present. Relative abundance is expressed as a fraction on a logarithmic scale (dot plots) or as a fraction in the linear scale (bar graphs).

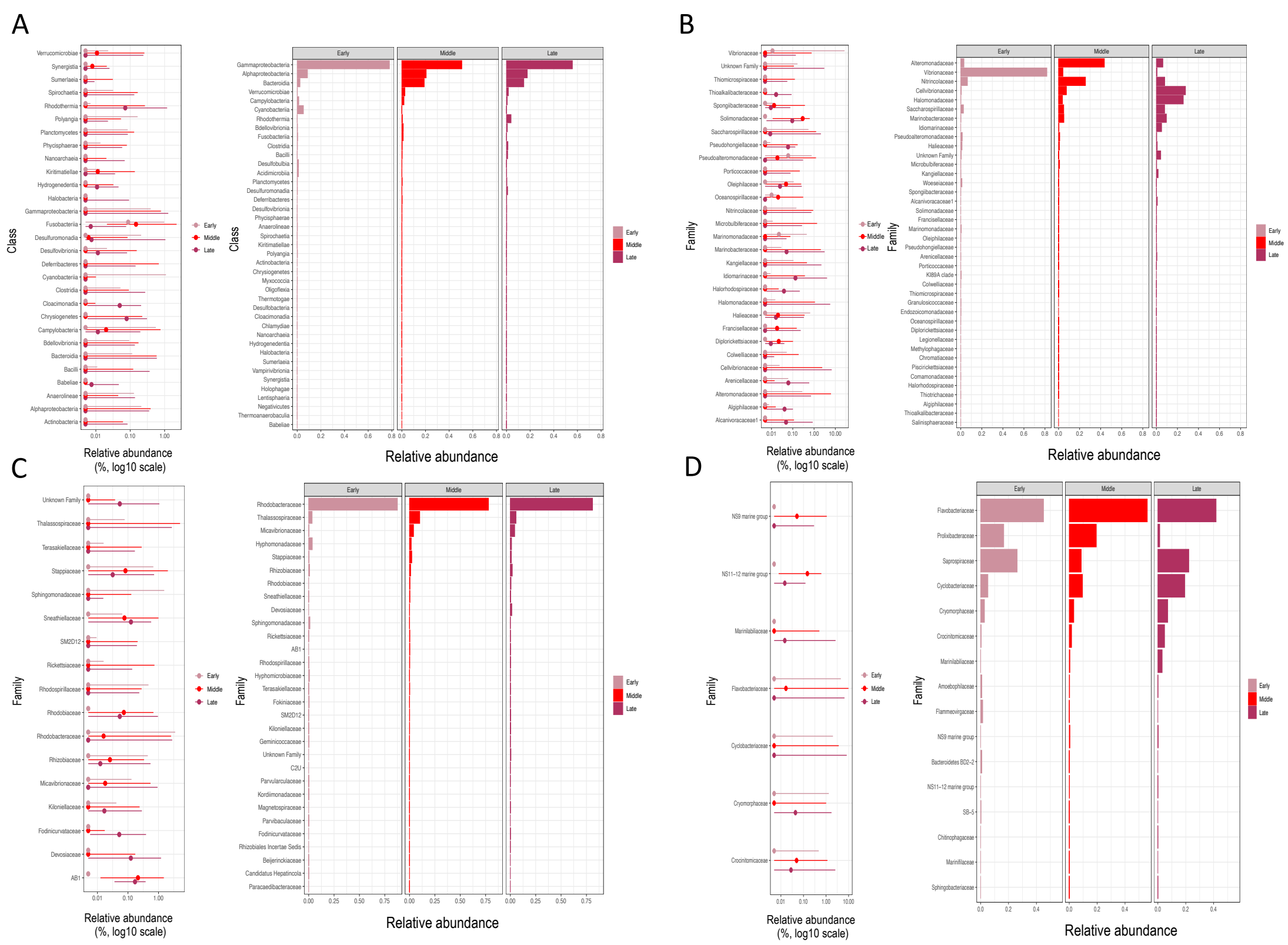

Supplementary Figure S6. Variation in relative abundance of the predicted functional profiles for the three phases identified in the decomposition process (early, middle, and late) and Kruskal-Wallis tests. The dot plot includes only functions that are significantly different based on Kruskal-Wallis tests ($p<0.05$). Dots represent medians and lines represent 95% confidence intervals. The bar graph represents all functions that are present. Relative abundance is expressed as a percentage (%), on a logarithmic scale (dot plot) or as a fraction (bar plot).

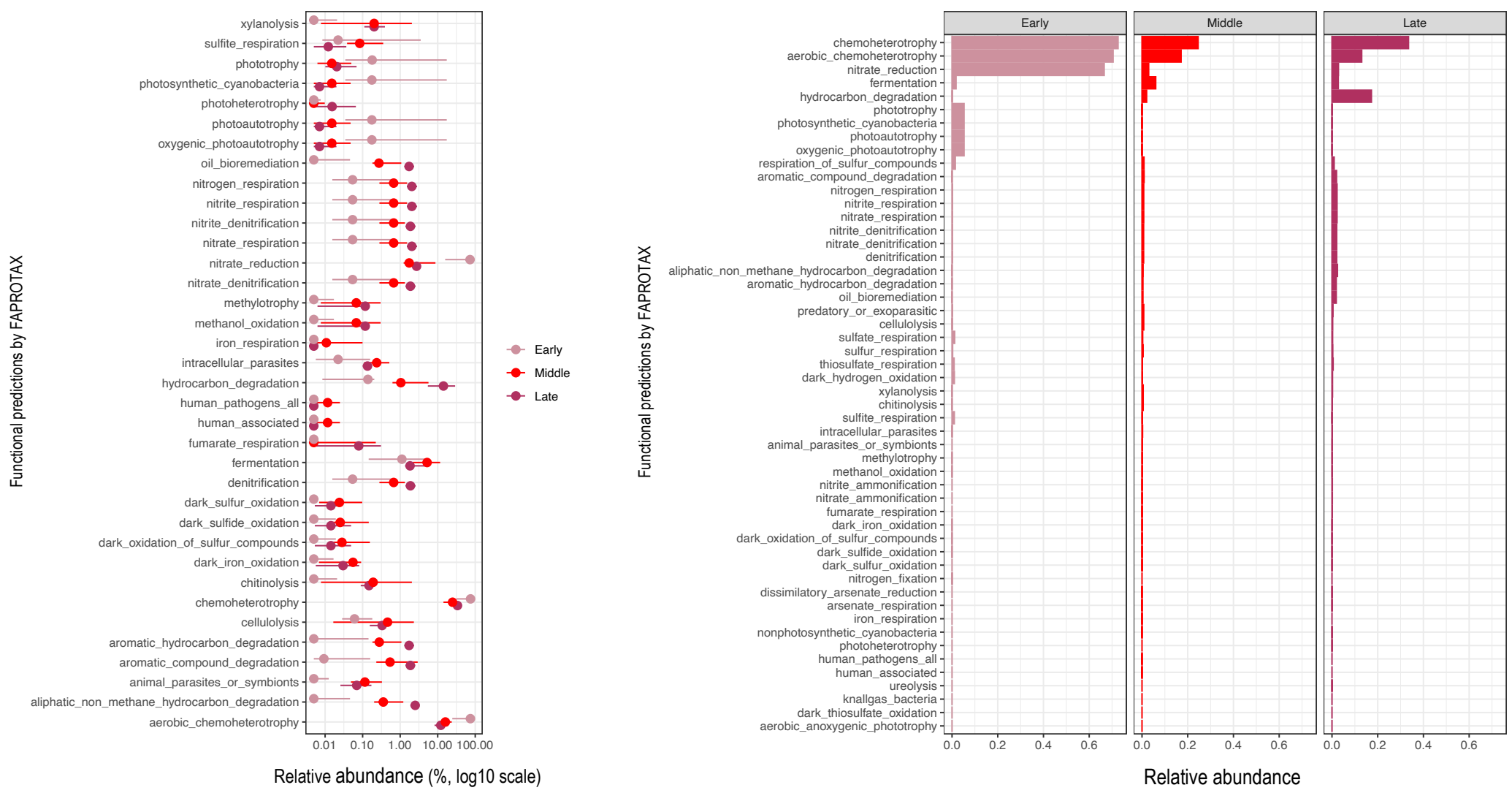

Supplementary Figure S7. Comparison of data for the isotopic signature of nitrogen ($\delta^{15}N$) of *Sargassum* thalli documented by Vázquez-Delfin et al. (2021), as a function of the variation in relative abundance of *S. natans* I and *S. fluitans* III for the populations that arrive to the Mexican Caribbean. Plot (A) compares the variation of $\delta^{15}N$ as a function of changes in relative abundance of *S. natans* I (in orange) and *S. fluitans* III (in green); and Plot (B) illustrates the strong negative collinearity in the abundance of both variants of *Sargassum* for the populations that arrive to the Mexican Caribbean.

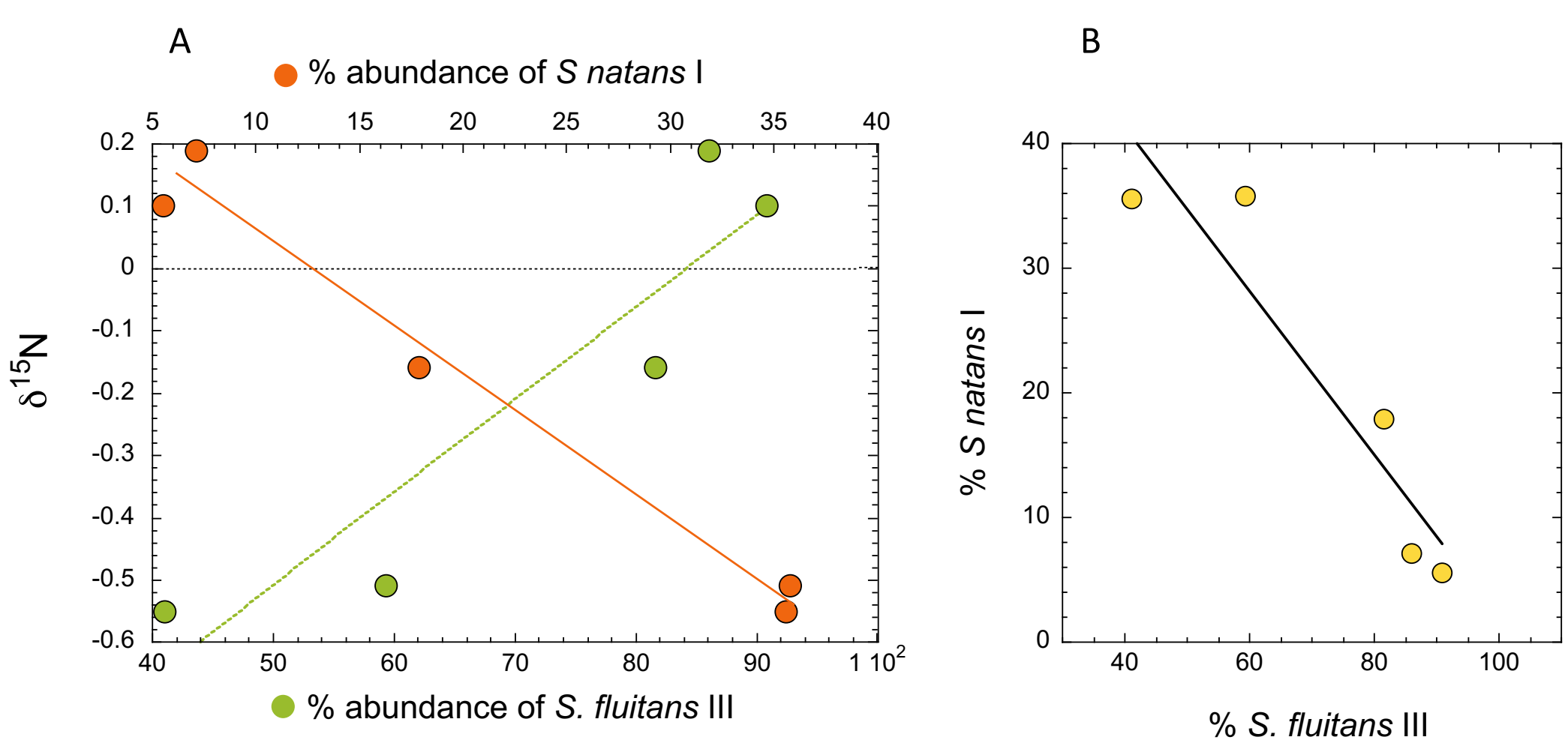

2. Supplementary Tables:

Table S1. Impact of *Sargassum* tides on oxygen dissolved in coastal waters:

Site	Year	Condition	Zone	Mean±SD (mg L^{-1})	Ranges (mg L^{-1})	Source
Puerto Morelos	Summer 2008	*Sargassum* absence	Coast	7.19 ± 1.29	5.25 – 9.15	Van tussenbroek et al., (2017)
Mahahual, Xahuayxol, Xcalak	2016-2017	Low influx years	Coast-backreef	9.74 ± 0.40	6.65 – 10.23	Cabanillas-Terán et al., (2025)
Punta Nizuc	Autuum 2019	*Sargassum* beaching	Near-shoreline (transparent water)	6.43		Saldaña-Fabela et al. (2019)
Punta Nizuc	Autuum 2019	*Sargassum* beaching	Coast	5.56 ± 1.79	1.29 – 8.01	Saldaña-Fabela et al. (2019)
Puerto Morelos	Summer 2015	*Sargassum* beaching	Coast	4.96 ± 0.15	4.66 – 5.25	Van tussenbroek et al., (2017)
Mahahual	Summer 2016	*Sargassum* beaching	Backreef	4.1 ± 0.34		Cabanillas-Terán et al., (2019)
Xcalak	Summer 2016	*Sargassum* beaching	Near-shoreline	4.8 ± 0.22		Cabanillas-Terán et al., (2019)
Mahahual	Summer 2016	*Sargassum* beaching	Near-shoreline	3.66 ± 0.42		Cabanillas-Terán et al., (2019)
Punta Nizuc	Autuum 2019	*Sargassum* beaching	Near-shoreline (brown light water)	3.5		Saldaña-Fabela et al. (2019)
Mahahual, Xahuayxol, Xcalak	2016-2017	High influx year	Coast-backreef	3.02 ± 1.8	0 – 7.15	Cabanillas-Terán et al., (2025)
Xahuayxol	Summer 2016	*Sargassum* beaching	Near-shoreline (brown tide)	2.42 ± 0.32		Cabanillas-Terán et al., (2019)
Xcalak, Mahahual, Xahuayxol	Autum 2018	*Sargassum* beaching	Near-shoreline (brown tide)	3.4 ± 0.77	2.62 ±0.16 – 4.16 ± 0.72	Rodríguez-Muñoz et al., (2021)

Puerto Morelos	Summer 2018	*Sargassum* beaching	Near-shoreline (brown tide)	2.9	1.9 – 4.2	Rodríguez-Martínez et al., (2019)
Mahahual	Summer 2016	*Sargassum* beaching	Leachetes	1.88 ± 0.37		Cabanillas-Terán et al., (2019)
Xcalak	Summer 2016	*Sargassum* beaching	Leachetes	1.01 ± 0.30		Cabanillas-Terán et al., (2019)
Xcalak, Mahahual, Xahuayxol	Autum 2018	*Sargassum* beaching	Leachetes	0.17 ± 0.12	0.05 ± 0.04 – 0.29 ± 0.41	Rodríguez-Muñoz et al., (2021)

References:

Cabanillas-Terán, N., Hernández-Arana, H. A., Ruiz-Zárate, M. Á., Vega-Zepeda, A., & Sanchez-Gonzalez, A. (2019). Sargassum blooms in the Caribbean alter the trophic structure of the sea urchin Diadema antillarum. PeerJ, 7, e7589.

Saldaña Fabela, P., Izurieta Dávila, J., Gómez Balandra, A., Martínez Jiménez, M., Bravo Inclán, L., Vázquez Bustos, C., & Huerto Delgadillo, R. (2019). Diagnóstico de calidad del agua y caracterización de las dos especies de sargazo que arriban a las costas de Cancún, Puerto Morelos y Playa del Carmen. Coordinación de tratamiento y calidad del agua subcoordinacion de hidrobiología y evaluación ambiental. Secretaria de Medio Ambiente y Recursos Naturales. Instituto Mexicano de Tecnología del Agua. TC1907.1. Diciembre 2019.

Rodríguez-Martínez, R. E., Medina-Valmaseda, A. E., Blanchon, P., Monroy-Velázquez, L. V., Almazán-Becerril, A., Delgado-Pech, B., Vásquez-Yeomans, L., Franscisco, V., & García-Rivas, M. C. (2019). Faunal mortality associated with massive beaching and decomposition of pelagic Sargassum. *Marine pollution bulletin*, *146*, 201-205.

Rodríguez-Muñoz, R., Muñiz-Castillo, A. I., Euán-Avila, J. I., Hernández-Núñez, H., Valdés-Lozano, D. S., Collí-Dulá, R. C., & Arias-González, J. E. (2021). Assessing temporal dynamics on pelagic Sargassum influx and its relationship with water quality parameters in the Mexican Caribbean. Regional Studies in Marine Science, 48, 102005.

Van Tussenbroek, B. I., Arana, H. A. H., Rodríguez-Martínez, R. E., Espinoza-Avalos, J., Canizales-Flores, H. M., González-Godoy, C. E., Barba-Santos, M. G., & Collado-Vides, L. (2017). Severe impacts of brown tides caused by Sargassum spp. on near-shore Caribbean seagrass communities. Marine pollution bulletin, 122(1-2), 272-281.

Table S2. Variation in nutrient concentration in coastal waters associated with *Sargassum* tides. TDN = Total dissolved nitrogen = Dissolved organic nitrogen (DON) + Dissolved inorganic nitrogen (DIN)). TPN = Total inorganic phosphorus = Dissolved organic phosphorus (DOP) + Dissolved inorganic phosphorus (DIP)). DIN = Dissolved inorganic nitrogen = $NH_4^+ + NO_3^- + NO_2^-$

Site (label)	Year	Condition	Zone	TDN (µM)	TDP (µM)	DIN±SE (µM)	DIP±SE (µM)	Source
Cancun	Summer-2019	High influx	Near shorline	109.32±7.24	0.89±0.04	41.26±37.64		Saldaña-Fabela et al. (2019)
Puerto Morelos	Summer-2019	High influx	Near shorline	206.06±94.41	5.55±4.05	13.95±7.7		Saldaña-Fabela et al. (2019)
Playa del Carmen	Summer-2019	High influx	Near shorline	110±6.96	0.99±0.15	14.47±1.18		Saldaña-Fabela et al. (2019)
Playa del Carmen	Summer-2019	*Sargassum* in descomposition	Coast	778.12±495	35.02±26.75	89.7±3.48		Saldaña-Fabela et al. (2019)
Tulum	Summer-2019	High influx	Near shorline	142.97	0.94	2.92		Saldaña-Fabela et al. (2019)
Tulum	Summer-2019	*Sargassum* in descomposition	Coast	255.68±0.41	12.26±1.29	18.35±1.41		Saldaña-Fabela et al. (2019)
Huache, Mx	Summer - 2017	Low influx	Mid-lagoon			18.25±1.47	0.82±0.10	Velázquez-Ochoa and Enríquez (2023)
Huache, Mx	Summer - 2018	High influx	Mid-lagoon			30.84±1.5	1.80±0.15	Velázquez-Ochoa and Enríquez (2023)
Huache, Mx	Summer - 2017	Low influx	Back-reef			26.24±4.4	0.93±0.10	Velázquez-Ochoa and Enríquez (2023)
Huache, Mx	Summer - 2018	High influx	Back-reef			31.20±1.34	1.95±0.10	Velázquez-Ochoa and Enríquez (2023)
Mahahual	Summer-2019	High influx	Near shorline	129.74	0.93	1.20		Saldaña-Fabela et al. (2019)
Mahahual	Summer-2019	*Sargassum* in descomposition	Coast	198.77	3.91	6.60		Saldaña-Fabela et al. (2019)
Xcalak	Summer-2019	High influx	Near shorline	130.21	0.76	1.11		Saldaña-Fabela et al. (2019)
Xcalak	Summer-2019	*Sargassum* in descomposition	Coast	422.40±254	23.66±21.06	12.28±1.9		Saldaña-Fabela et al. (2019)

References

Saldaña Fabela, P., Izurieta Dávila, J., Gómez Balandra, A., Martínez Jiménez, M., Bravo Inclán, L., Vázquez Bustos, C., & Huerto Delgadillo, R. (2019). Diagnóstico de calidad del agua y caracterización de las dos especies de sargazo que arriban a las costas de Cancún, Puerto Morelos y Playa del Carmen. Coordinación de tratamiento y calidad del agua subcoordinacion de hidrobiología y evaluación ambiental. Secretaria de Medio Ambiente y Recursos Naturales. Instituto Mexicano de Tecnología del Agua. TC1907.1. Diciembre 2019.

Velázquez-Ochoa, R., & Enríquez, S. (2023). Environmental degradation of the Mexican Caribbean reef lagoons. *Marine Pollution Bulletin*, *191*, 114947.

Table S3. Variation of toxic gas emissions associated with *Sargassum* tides. CH_4 = methane, H_2S = Hydrogen sulfide. N.D. = no data available.

Site	Date	Condition	CH_4 ± SD (ppm)	H_2S ± SD (ppm)	Source
Cancun	Summer 2019	*Sargassum* beached	603	0 ± 0	Saldaña-Fabela et al., (2019)
Puerto Morelos	Summer 2019	*Sargassum* beached	301.5 ± 80.5	0 ± 0	Saldaña-Fabela et al., (2019)
Puerto Morelos	Summer 2022	*Sargassum* piled	N. D.	21.27 ± 10.30	Rodríguez-Martínez et al., (2024)
Puerto Morelos	Winter - spring 2022	*Sargassum* piled	N. D.	37.14 ± 15.40	Rodríguez-Martínez et al., (2024)
Puerto Morelos	Summer 2023	*Sargassum* piled	N. D.	16.48 ± 8.16	Rodríguez-Martínez et al., (2024)
Puerto Morelos	Summer 2022	Ground digging	N. D.	5.89 ± 12.59	Rodríguez-Martínez et al., (2024)
Puerto Morelos	Winter - spring 2022	Ground digging	N. D.	8.24 ± 20.19	Rodríguez-Martínez et al., (2024)
Puerto Morelos	Summer 2023	Ground digging	N. D.	5.73 ± 14.82	Rodríguez-Martínez et al., (2024)
Puerto Morelos	Summer 2022	Ground 10m distance	N. D.	0.16 ± 0.46	Rodríguez-Martínez et al., (2024)
Puerto Morelos	Winter - spring 2022	Ground 10m distance	N. D.	0.07 ± 0.27	Rodríguez-Martínez et al., (2024)
Puerto Morelos	Summer 2023	Ground 10m distance	N. D.	0.04 ± 0.18	Rodríguez-Martínez et al., (2024)
Puerto Morelos	Summer 2025	Sargassum beached	N. D.	2.3 ± 2.7	Rodríguez-Martínez et al., (2026)
Playa del Carmen	Summer 2019	*Sargassum* beached	290.5 ± 228.5	0 ± 0	Saldaña-Fabela et al., (2019)
Playa del Carmen	Summer 2025	*Sargassum* beached		0.8 ± 2,1	Rodríguez-Martínez et al., (2026
Tulum	Summer 2019	*Sargassum* beached	144.65 ± 108.35	0 ± 0	Saldaña-Fabela et al., (2019)
Mahahual	Summer 2019	*Sargassum* beached	294 ± 105.5	148.35 ± 53.44	Saldaña-Fabela et al., (2019)
Othon P. Blanco	Summer 2025	*Sargassum* beached	N. D.	1.9 ± 2,6	Rodríguez-Martínez et al., (2026)
Xcalak	Summer 2019	*Sargassum* beached	242	0	Saldaña-Fabela et al., (2019)
Xcalak	Summer 2019	Leaches	1154	187	Saldaña-Fabela et al., (2019)

References

Rodríguez-Martínez, R. E., Reali, M. Á. G., Torres-Conde, E. G., & Bates, M. N. (2024). Temporal and spatial variation in hydrogen sulfide (H2S) emissions during holopelagic Sargassum spp. decomposition on beaches. *Environmental Research*, *247*, 118235.

Rodríguez-Martínez, R. E., Gómez-Reali, M. A., Rosales-Fuerte, S., Pech Figueroa, J., Costa, M., & MM, V. (2026). Assessment of hydrogen sulfide exposure in shoreline cleanup workers during the 2025 Atlantic Sargassum bloom. *Harmful Algae*, 103092.

Saldaña Fabela, P., Izurieta Dávila, J., Gómez Balandra, A., Martínez Jiménez, M., Bravo Inclán, L., Vázquez Bustos, C., & Huerto Delgadillo, R. (2019). Diagnóstico de calidad del agua y caracterización de las dos especies de sargazo que arriban a las costas de Cancún, Puerto Morelos y Playa del Carmen. Coordinación de tratamiento y calidad del agua subcoordinacion de hidrobiología y evaluación ambiental. Secretaria de Medio Ambiente y Recursos Naturales. Instituto Mexicano de Tecnología del Agua. TC1907.1. Diciembre 2019.
